# Childhood appendectomy is linked with higher digestive, respiratory, and genitourinary disease risk but lower inflammatory bowel disease risk

**DOI:** 10.1093/emph/eoag011

**Published:** 2026-06-11

**Authors:** Sean G Byars, Stephen C Stearns, Jacobus J Boomsma

**Affiliations:** School of Translational Medicine, The Alfred Centre, Monash University, Melbourne, Australia; Department of Biology, University of Copenhagen, Copenhagen, Denmark; Department of Ecology and Evolutionary Biology, Yale University, New Haven, CT, USA; Department of Biology, University of Copenhagen, Copenhagen, Denmark

**Keywords:** appendectomy, appendix, long-term disease risk, microbiome, immune development, Nationwide cohort study

## Abstract

**Background and objectives:**

Appendectomy is a common pediatric procedure generally considered safe beyond perioperative risks. However, the appendix may support gut biofilm maintenance and immune function, raising questions about potential long-term health consequences of its removal during childhood.

**Methodology:**

We examined associations between appendectomy before age 12 and subsequent risk of 25 disease outcomes between ages 12 and 30, using a population-based cohort of up to 1 071 086 children born in Denmark between 1979 and 1999 and followed through linked national registers until 2009. Stratified Cox regression models compared surgically treated individuals with matched controls without significant pre-surgical health differences. Analyses adjusted for pregnancy complications, parental disease history, birth weight, Apgar score, sex, and socioeconomic factors.

**Results:**

Childhood appendectomy was associated with increased risk of several disease categories, particularly digestive [relative risk (RR) 1.45–1.64], respiratory (RR 1.20–1.73), and genitourinary disorders (RR 1.30–1.72). In contrast, inflammatory bowel disease (IBD) risk was reduced (RR 0.58). Absolute risk increases were most notable for digestive (up to 2.81%) and respiratory diseases (up to 4.89%), suggesting measurable population-level associations.

**Conclusions and implications:**

Childhood appendectomy is associated with altered long-term disease risk. These findings support evidence that the appendix contributes to digestive and immune function. Although the reduced risk of IBD may represent a beneficial association, our results suggest that the appendix is not functionally redundant, particularly during immune development in childhood.

## INTRODUCTION

Continued debate has surrounded the function of the appendix for more than a century. Historically regarded as a vestigial appendage, the appendix has more recently been proposed to act as a bacterial reservoir that may help re-inoculate the gut after diarrheal disease [1]. Because severe diarrheal disease was more common in ancestral environments, this proposed function has sometimes been interpreted as having limited relevance in modern settings, where sanitation and medical care have reduced the frequency and severity of enteric infections. However, the adaptive significance of the appendix has become increasingly appreciated [2], and its potential long-term importance for human health remains unresolved. Appendectomy can be life-saving in acute appendicitis, but it is also sometimes performed when appendicitis is not present [3]. Research to date has largely focused on short-term surgical risks, such as post-operative bleeding [4], rather than on whether appendectomy may be associated with longer-term health risks, particularly when performed in childhood, a period of ongoing immune maturation and acquisition of immunological memory [5–7]. We therefore asked whether appendectomy performed in childhood is associated with disease risk up to 30 years of age.

Historically, the function of the appendix has intrigued scientists and physicians, from discussions by early anatomists [8, 9] to Darwin’s argument in ‘The Descent of Man’ [10] that the organ was too small to contribute meaningfully to digestion. Over the last century, evidence has accumulated to challenge the view that the appendix is vestigial. For example, the appendix contains substantial gut-associated lymphoid tissue [11], suggesting an immune function. Comparative studies have shown that the appendix is present in more species than previously thought, including lorises, lemurs, monkeys, and all apes [12], and that it probably evolved independently in at least three major mammalian clades, including primates, glires, and marsupials [13]. The appendix has also been proposed to act as a ‘safe house’ for beneficial bacteria that support intestinal biofilms [14, 15]. These biofilms may contribute to digestion and facilitate re-inoculation of the gut after diarrheal disease [1, 16], with appendectomy also being associated with enduring changes in gut fungal communities [17]. Although the likely functions of the appendix are now better understood, it is still commonly removed on the assumption that long-term side effects are unlikely [1]. This assumption requires further empirical evaluation.

**Figure 1:**
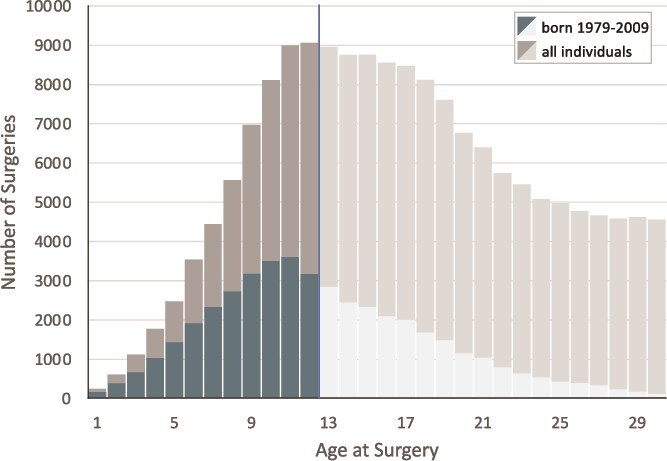
Age at appendectomy for 1 757 770 Danish residents born between 1979 and 2009 (blue colours) compared to all individuals living in Denmark during that time (grey colours), and the selected appendectomy observation window of 0–12 years; the cut-off for inclusion as appendectomy cases (dark blue bars) was chosen because it represents childhood and early development, captures common early ages in which many of these surgeries are performed, and maximized the number of years available for follow-up after appendectomy; individuals with appendectomy beyond the 12-year observation end point (blue vertical line) were not included as either cases or controls.

The growing recognition of the microbiota’s role in human health has increased understanding of how dysbiosis can contribute to immune, metabolic, and neurological disorders [18, 19]. For example, reduced gut bacterial diversity has been associated with inflammatory bowel disease (IBD) [20], childhood asthma [21], and obesity [22]. In addition to its proposed role in gut re-inoculation, the appendix—a blind sac extending from the caecum—contains lymphatic nodules and mucosa-lined crypts that may facilitate immune homeostasis and protect microbial inhabitants from competitors [17, 18]. Removal of the appendix could therefore plausibly have long-term health consequences in modern environments. Several recent studies in adult populations support this possibility, reporting altered risk of ulcerative colitis (UC), Crohn’s disease [23, 24], ischemic heart disease [25], certain infections [26], and other disease outcomes after appendectomy [27]. These findings motivate investigation of a broader range of diseases at younger ages, where evidence remains limited. The appendix is largest during childhood, and the number and size of its lymphoid nodules decrease with age, suggesting that its function may be particularly important early in life [28]. Given that appendectomy is commonly performed in childhood and adolescence (Fig. 1), it is important to assess whether appendectomy at these ages is associated with long-term disease risk.

Here, we used linked Danish health registry data for almost 1.1 million children followed from birth up to 30 years of age to address three questions: (i) Is appendectomy performed in the first 12 years of life associated with subsequent disease risk up to age 30? (ii) Are associations stronger for particular disease categories, especially diseases involving the immune or digestive systems? (iii) Where associations are observed, are they large enough in absolute terms to represent measurable population-level differences in disease burden?

## METHODOLOGY

We estimated associations between appendectomy performed in the first 12 years of life and subsequent disease risk from ages 12 to 30 years. This exposure window captures one of the most common age ranges for appendectomy, both generally [29] and in our sample (Fig. 1), while allowing sufficient follow-up after childhood exposure. To reduce reverse causation and improve comparability between appendectomy cases and controls, we excluded individuals with relevant disease diagnoses before age 12 and assessed outcomes only after this age. Because Danish health registries record hospital-diagnosed diseases from birth onwards, we were able to assess whether appendectomy cases and controls differed in early general morbidity before follow-up. These supplementary analyses found no material differences in early-life general morbidity between cases and controls, supporting the comparability of the groups before outcome assessment (see Supplementary Methods for full details). We used Cox regression models stratified by sex, birth cohort, birth season, and demographic parity, and adjusted for an additional 18 covariates. We also performed power analyses to confirm that sample sizes were adequate for each disease group.

### Study sample from Danish registries

We used data from the Danish Birth Registry of ~1.1 million individuals born as singletons between 1979 and 1999 whose health was evaluated up to 2009 (sample sizes in Supplementary Table S1). This design allowed us to determine whether appendectomy occurred between birth and 12 years of age and to assess subsequent disease outcomes from age 12 up to age 30, with up to 18 years of follow-up after age 12.

The analytic sample included appendectomy cases and controls without diseases diagnosed between birth and 12 years of age, and who did not undergo appendectomy after age 12 and before age 30. Individuals born before 1979 were excluded because complete diagnostic histories from birth were not available, as Danish electronic health data are unavailable before 1977–78. Follow-up was limited to 2009 because data beyond this date were not available at the time of data request. Figure 1 shows appendectomies by age in Denmark, and Supplementary Table S1 provides disease-specific sample sizes used in the analyses.

This design reduces the potential for reverse causation and bias due to incomplete early-life diagnostic data, while maintaining sufficient sample size and statistical power to estimate differences in disease risk between appendectomy cases and controls. Individuals included in the analyses had complete data for all 22 covariates considered and were within normal ranges for quantitative covariates, including birth weight (1850–5400 g), gestation length (30–42 weeks), and paternal (15–60 years) and maternal age (15–46 years) at birth.

Other variables included in the analyses were derived from linked Danish registries using unique personal identification numbers, de-identified from Central Person Register numbers. These registries included the Danish Birth Registry, containing pregnancy, birth, and pedigree information; the Danish National Patient Registry, containing nationwide hospital admission and International Classification of Diseases (ICD) diagnosis data; the Danish Psychiatric Registry, containing psychiatric diagnoses from inpatient admissions; and civil registration and cause-of-death registries, containing information on death, migration, socioeconomic status, and other demographic variables. The free health system in Denmark reduces socioeconomic barriers to healthcare. Its comprehensive linked registry infrastructure provides longitudinal health, life history, and socioeconomic information for the individuals analysed. These data also extend into the parental generation, allowing adjustment for early-life and familial covariates. Characteristics of the study population are shown in Table 1.

**Table 1:** Characteristics of the initial study sample (*n* = 1 757 770) for appendectomy and control groups.

	Appendectomy	Control	Total
Sample size (*n*)	44 496	1 713 274	1 757 770
Paternal age (*μ*)	30.0 (5.7)	31.3 (5.7)	31.2 (5.7)
Maternal age (*μ*)	27.1 (4.8)	28.5 (4.8)	28.4 (4.9)
Gestation length, weeks (*μ*)	39.7 (1.6)	39.7 (1.6)	40.0 (1.6)
Maternal bleeding (%)	7.9 (0.2)	7.3 (0.2)	7.4 (0.2)
Foetal oxygen deprivation (%)	0.17 (0.04)	0.09 (0.03)	0.09 (0.03)
Pregnancy oedema (%)	1.35 (0.12)	0.79 (0.09)	0.82 (0.09)
APGAR 5 score 1–10 (*μ*)	9.8 (0.5)	9.8 (0.6)	9.8 (0.6)
Birth weight (g) (*μ*)	3457 (537)	3499 (541)	3495 (543)
Pre-existing hypertension (%)	0.16 (0.04)	0.33 (0.06)	0.32 (0.06)
Pre-existing diabetes (%)	0.29 (0.05)	0.39 (0.06)	0.39 (0.06)
Previous induced abortion (%)	18.7 (0.4)	18.2 (0.3)	18.3 (0.3)
Previous spontaneous abortion (%)	15.6 (0.3)	14.2 (0.3)	14.3 (0.3)
Education, combined total yrs (*μ*)	24.8 (4.8)	25.6 (4.7)	25.5 (4.8)
Income, combined average Dkr. (*μ*)	407 502 (169 955)	362 241 (172 797)	363 188 (172 296)
Nationality, 0 = Danish, 1 = other (%)	3.33 (0.18)	6.89 (0.25)	6.83 (0.25)
Region in Denmark most lived (%)			
Hovedstaden	25.8 (0.4)	28.7 (0.4)	28.5 (0.4)
Sjælland	15.3 (0.3)	14.5 (0.3)	14.7 (0.3)
Syddanmark	25.2 (0.4)	22.4 (0.4)	22.3 (0.4)
Midtjylland	22.6 (0.4)	23.7 (0.4)	23.6 (0.4)
Nordjylland	10.9 (0.3)	10.5 (0.3)	10.6 (0.3)
Birth year (*μ*)	1986 (6)	1994 (8)	1993 (8)
Birth season, months 1–12 (*μ*)	6.3 (3.3)	6.4 (3.3)	6.4 (3.3)
Sex, 0 = male, 1 = female (%)	46.3 (0.5)	48.6 (0.5)	48.7 (0.5)
Demographic parity (%)			
First born	45.3 (0.5)	44.1 (0.5)	44.3 (0.5)
Second born	37.1 (0.4)	37.3 (0.4)	37.3 (0.4)
Third born	13.2 (0.3)	13.8 (0.3)	13.7 (0.3)
Fourth (or higher born)	4.18 (0.2)	4.7 (0.21)	4.67 (0.21)

Numbers, means, percentages, and (SDs) are provided. This includes the study sample before any exclusions (i.e. outliers, missing values) were applied. For final sample sizes, see Table S1.

### Appendectomy procedure codes

Appendectomy was identified using surgical procedure codes from Statistics Denmark operation classifications up to 1996 and the Nordic Medico-Statistical Committee Classification of Surgical Procedures from 1996 onwards. Appendectomy codes included 4300, JEA00, JEA01, and JEA10.

### Disease ICD groups

Because immune dysfunction or dysbiosis could plausibly be associated with a broad range of organismal processes, we examined 25 disease groups, including diseases linked to the immune system, such as infections and allergies; digestive tract diseases, including IBD; respiratory diseases; and other major disease categories, including circulatory, nervous system, endocrine, genitourinary, musculoskeletal, neoplastic, and psychiatric disorders. A full list of disease groups and ICD codes is provided in Supplementary Table S2A. In Denmark, ICD-8 was used from 1969 to 1993 and ICD-10 from 1994 onwards.

### Covariates adjusted for

Health and socioeconomic covariates adjusted for in analyses included: binary variables for maternal pre-existing conditions (Supplementary Table S2B, ICD codes) including hypertension (primary or secondary hypertension, hypertensive heart, or renal disease), diabetes (i.e. type-I or type-II, malnutrition-related, other or unspecified), previous spontaneous or induced abortions; maternal pregnancy-related variables including gestation length (in weeks), and binary variables for the presence of maternal bleeding (i.e. haemorrhage, placenta praevia), foetal oxygen deprivation (i.e. hypoxia, asphyxia), and pregnancy oedema.

Parental variables included a binary indicator for whether either parent had ever received a diagnosis within the same disease group as the child, to account for familial transmission, the total number of years of education summed across both parents, and the average income across the study period summed across both parents. Birth-related variables included birth weight (in g), birth season (calendar month, 1 to 12), birth cohort (3-yearly between 1979 and 1999 accounting for possible changes in diagnostic criteria over time), and the APGAR5 (Appearance, Pulse, Grimace, Activity, Respiration) score of 1–10 (maximally 2 points for each category) given to babies shortly after birth ranging from poor to excellent health. Other child-related variables included sex (0 = male, 1 = female), nationality (0 = Danish national, 1 = immigrant), parity, and the region within Denmark (Hovedstaden = Copenhagen Area, Sjælland, Syddanmark, Midtjylland, and Nordjylland) in which a child had resided for the longest period to account for possible regional differences in diagnosis and healthcare use.

### Statistical design and analysis

Cox regression models were used to estimate relative risks (RRs) for each of the 25 disease outcomes between ages 12 and 30 years, using age as the timescale. The primary exposure was appendectomy before age 12. Models were stratified by sex, birth cohort, birth season, and demographic parity to satisfy proportional hazards assumptions, and adjusted for the additional covariates described above. Cox regression assumptions were assessed before final model specification.

To reduce the likelihood of false-negative results due to inadequate power, we performed power analyses using the powerSurvEpi package in R v3.2.1. This package estimates the minimum sample size needed to detect an effect of a specified size, based on the postulated risk ratio, variance of the main exposure variable, proportion of individuals diagnosed with the disease, desired power, and type I error rate. We used the observed RRs for the focal disease outcomes as the postulated risk ratios. All disease groups met these thresholds. To account for multiple testing across the 25 disease outcomes, we adjusted the *P*-values using the Benjamini–Hochberg false discovery rate procedure. Bonferroni-adjusted significance thresholds are also reported as a more conservative sensitivity criterion.

**Figure 2:**
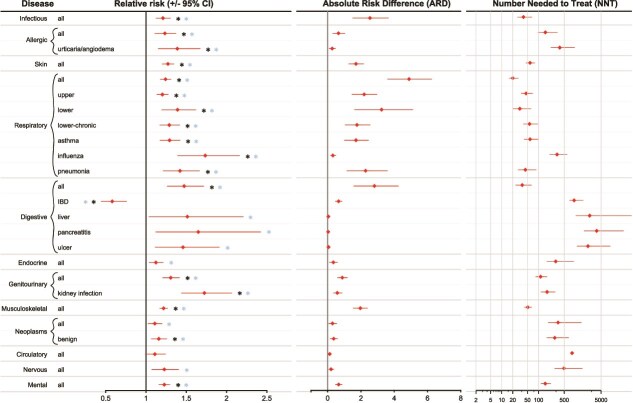
Risk of disease up to age 30 years after removal of the appendix in the first 12 years of life; relative risks (RRs) and 95% confidence intervals (CIs) were estimated from Cox regression models and represent the risk of each disease up to age 30 years among individuals who underwent appendectomy relative to controls who did not undergo appendectomy; statistical significance after correction for 25 disease tests is indicated by stars next to the upper confidence interval for each disease: blue stars indicate significance after Benjamini–Hochberg false discovery rate correction, and black stars indicate significance after Bonferroni correction; where both criteria were met, both stars are shown; absolute risk differences (ARDs) and 95% CIs were estimated as ARD = 100 × CR × (1−RR), where CR is the disease risk in the control sample and RR is the relative risk among individuals post-surgery compared with controls; number needed to treat values, interpreted as number needed to benefit or harm depending on the direction of association, were estimated as NNT = 100/ARD; in the ARD and NNT panels, values are displayed on an absolute scale to aid visualization, with the direction of association inferred from the RR panel: RR > 1 indicates increased risk and NNT-harm, whereas RR < 1 indicates reduced risk and NNT-benefit; only one disease showed reduced risk after appendectomy; accompanying RR, CR, ARD, and NNT benefit/harm values are provided in Supplementary Table S3; abbreviations: IBD, inflammatory bowel disease; ulcer, ulcerative colitis.

To aid interpretation of RRs in terms of population-level effect size [30] and clinically interpretable measures [31], we used RRs derived from Cox regression models and disease prevalence in controls, defined as control risk (CR), to estimate absolute risk differences (ARDs) and number needed to treat (NNT) values. ARDs were estimated as:


$$ARD=100\times CR\times \left(1- RR\right)$$

where positive values indicate increased absolute risk after appendectomy and negative values indicate reduced absolute risk. NNT values were estimated as:


$$NNT=100/ ARD$$

and interpreted as number needed to harm when ARD was positive and number needed to benefit when ARD was negative. For graphical presentation in Fig. 2, ARD and NNT values were displayed on an absolute scale, with the direction of association inferred from the corresponding RR estimate. Full signed RR, CR, ARD, and NNT benefit/harm values are provided in Supplementary Table S3.

## RESULTS

Childhood appendectomy was associated with altered risk across multiple disease groups up to age 30 years (Fig. 2, Supplementary Table S3). Most associations were in the direction of increased RR, although IBD showed reduced RR after appendectomy. These patterns suggest that appendectomy in childhood is associated with later differences in disease burden, with the magnitude of absolute risk depending on the baseline prevalence of each disease.

### Increased relative risk for digestive, genitourinary, and respiratory diseases

Disease risk was significantly elevated for 23 out of 25 disease groups after Benjamini–Hochberg false discovery rate correction, with the largest RRs observed for digestive, genitourinary, and respiratory disease groups (Fig. 2, Supplementary Table S3). Appendectomy was associated with nearly doubled RR of kidney infections [RR = 1.72, 95% confidence interval (CI) = 1.44–2.06] and influenza (RR = 1.73, 95% CI = 1.39–2.16), and with large increases in RR for all digestive (RR = 1.47, 95% CI = 1.26–1.71), pneumonia (RR = 1.42, 95% CI = 1.21–1.66), lower respiratory disease (RR = 1.39, 95% CI = 1.19–1.61), and urticaria/angioedema (RR = 1.38, 95% CI = 1.15–1.67).

Risks for liver disease, pancreatitis, and UC were also notable, with RRs ranging from 1.45 to 1.64 and significant after correction for multiple testing. The RRs often, but not always, corresponded to small changes in absolute risk and relatively large numbers needed to treat to prevent an additional harmful case (e.g. kidney infections: ARD = 0.58%, NNT-harm = 171). This implies that the effects of childhood appendectomy on many diseases in the first 30 years of life were significant but small at the population level.

### Association of appendectomy with lower inflammatory bowel disease risk

In contrast to increased risks observed for most disease groups, appendectomy was associated with decreased RR (RR = 0.58, 95% CI = 0.44–0.75, Fig. 2) of IBD, suggesting a protective effect for IBD later in life. However, the effect is modest at the population level with a small absolute risk reduction (ARD = −0.11%) and a high estimated NNT value of 926; in other words, ~926 appendectomies would be associated with one fewer IBD diagnosis by age 30 in this population. We found an increased risk for UC, a form of IBD included under that group in our analysis. We did not examine Crohn’s disease as our power analyses (above) indicated that we did not have sufficient sample size (due to the relative rarity in our sample) to robustly test that association.

### Absolute risk increases for common diseases

For common diseases such as those in the upper and lower respiratory tracts and also in broader disease categories such as all infectious or all respiratory diseases, even modest increases in RR (i.e. RR ranging from 1.20 to 1.39) resulted in relatively large increases in absolute disease risk (i.e. ARD increases of 2.19% to 4.89%) and relatively low NNT values (i.e. NNT below 50), which were driven by their high prevalence in the population (i.e. CR = 8.2% to 20.2%). For example, appendectomy was associated with significantly increased risk (21%) of all infectious diseases (RR = 1.21, 95% CI = 1.12–1.30, Supplementary Table S3). Because these diseases are relatively common (i.e. CR = 12%), this translates to an absolute risk increase of 2.54% and an NNT of only 39; in practical terms, this means that 39 appendectomies would be associated with one additional infectious disease diagnosis by age 30.

### Risks associated with covariates suggest complex disease aetiologies

**Figure 3:**
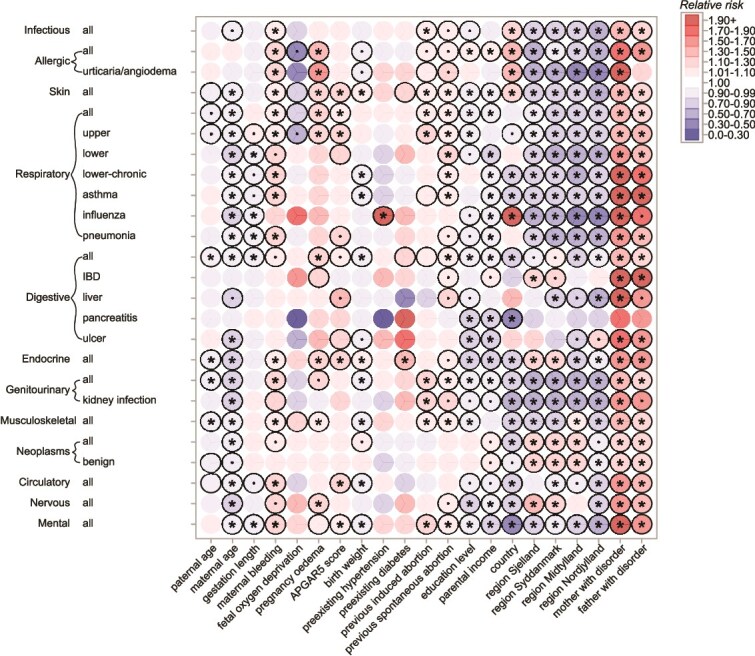
Disease risk patterns for covariates across disease categories; relative risks (RRs) were estimated from Cox regression models for disease outcomes within the first 30 years of life; covariates are shown on the horizontal axis and disease categories on the vertical axis; circle colour indicates the direction and magnitude of association, with blue representing decreased RR and red representing increased RR; the accompanying colour key shows the approximate RR range corresponding to each colour; for each disease–covariate combination, a black circle outline indicates nominal statistical significance before multiple-testing correction, defined as unadjusted *P* < .05; symbols within circles indicate associations that remained significant after correction for 25 disease tests: a black point indicates significance after Benjamini–Hochberg false discovery rate correction, and a black star indicates significance after Bonferroni correction; where Bonferroni significance was reached, the star is shown instead of the FDR point; disease risks for the Danish region of residence are relative to Hovedstaden, the Copenhagen region; abbreviations: IBD, inflammatory bowel disease; ulcer, ulcerative colitis.

Several covariates were associated with disease risk across many outcomes (Fig. 3, Supplementary Table S4), highlighting the importance of accounting for parental, perinatal, socioeconomic, and geographical factors when estimating associations between appendectomy and later disease risk. Some of the strongest covariate associations involved whether a mother or father had previously received a diagnosis within the same disease group as the child. Large RRs were observed for mental disorders (RR range = 1.60–2.01), asthma (RR range = 2.12–2.14), liver disease (RR range = 1.60–2.18), and IBD (RR range = 3.51–3.55). These patterns were similar for maternal and paternal disease history, consistent with an important familial component in these disease categories.

Other consistent covariate associations included maternal age and maternal bleeding during pregnancy. Higher maternal age at birth was associated with reduced risk for 21 (84%) of the 25 disease groups (Fig. 3), an effect that was largest in UC (RR = 0.82), kidney infections (RR = 0.84), endocrine diseases (RR = 0.85), and nervous system (RR = 0.87) diseases. In comparison, higher paternal age was only associated with a decreased risk in 6 (24%) of the 25 disease groups. Maternal bleeding during pregnancy was associated with increased risks for 18 of 25 diseases (RR ranging from 1.04 to1.22). These findings illustrate the general importance of parental factors in offspring disease risk.

Geographical and migration-related covariates also showed variation in association direction across disease outcomes. For example, the country variable categorizing whether individuals were Danish born (compared to immigrant) showed that being born in Denmark was associated with lower risk of 14 diseases (Fig. 3) and this tendency was lowest for mental disorders (RR = 0.49), pancreatitis (RR = 0.38), and kidney infections (RR = 0.52). Conversely, being Danish born was associated with increased risk of five diseases, including allergies (allergic all RR = 1.31, urticaria/angioedema RR = 1.41), skin diseases (RR = 1.24), infections (RR = 1.11), and influenza (RR = 2.06).

Socioeconomic covariates showed similarly heterogeneous associations. For example, higher parental education and income were associated with decreased risk for many diseases in children but increased risk of all allergic diseases (RR = 1.04), IBD (RR = 1.04), and cancer (RR = 1.01–1.02). Spatial patterns of disease risk were also important and varied, suggesting potential regional differences in disease risk (in comparison to Hovedstaden, i.e. Copenhagen Area). For example, many diseases, including mental disorders (RR ranged from 0.55 to 0.91), were less frequent in all other regions than Hovedstaden, but three regions (Sjælland, Syddanmark, Nordjylland) had higher risk of cancer than Hovedstaden (RR ranged from 1.10 to 1.17).

## DISCUSSION

We investigated whether childhood appendectomy was associated with subsequent risk of 25 disease outcomes up to 30 years of age in Denmark. Relative risks for many diseases were modestly but significantly increased after appendectomy, whereas IBD showed reduced RR. For common outcomes, particularly respiratory and infectious diseases, small changes in RR translated into larger ARDs. These findings support the hypothesis that the appendix may contribute to long-term immune and digestive function, potentially through effects on gut-associated lymphoid tissue, microbial biofilms, or broader host–microbiome interactions.

Surgeons are generally well aware of short-term risks before and after appendectomy in adults [32–35] and children [36], but long-term disease associations after childhood appendectomy have been less comprehensively investigated. Some previous studies have had substantial sample sizes, including a meta-analysis reporting reduced risk of UC after appendectomy [37], but few have combined large-scale population coverage, broad disease outcome assessment, early-life follow-up, and adjustment for extensive parental, perinatal, socioeconomic, and demographic covariates. Using Danish national health registries allowed us to examine disease risk after childhood appendectomy while accounting for a wide range of potential confounders. Appendectomy was associated with almost doubled RR of kidney infections and influenza, and increased risks were also observed for digestive, respiratory, and allergic diseases. Previous studies of childhood appendectomy have reported increased risk of adult mood and anxiety disorders after appendectomy performed before age 14 [38], acute myocardial infarction after appendectomy before age 20 [39], and cancer [40, 41]. These findings are broadly consistent with the increased risks we observed for mental disorders and neoplasms. Our findings also align with studies reporting associations between adult appendectomy and individual disease risks [23, 25, 42, 43].

In contrast to most disease outcomes, IBD risk was reduced after childhood appendectomy. Previous studies of appendectomy and subsequent IBD have been inconsistent, reporting increased [23, 44], decreased [37, 45], or mixed risk patterns [46–50]. Studies reporting mixed results often show increased short-term risk after appendectomy followed by no association or reduced risk over longer follow-up [47–50]. These inconsistencies may reflect differences in population structure, follow-up time, sample size, covariate adjustment, diagnostic definitions, and whether UC, Crohn’s disease, or IBD overall was examined. The reduced IBD risk observed here also complicates a simple beneficial-reservoir interpretation of appendix function. Several mechanisms could plausibly contribute to reduced IBD risk after appendectomy, although the evidence remains unresolved. The appendix contains abundant gut-associated lymphoid tissue and microbial biofilms, and may influence mucosal immune regulation and host–microbiome interactions. Because IBD involves dysregulated intestinal immune responses to microbial and environmental stimuli, appendectomy could plausibly alter immune or microbial pathways relevant to disease initiation or progression. However, this association may also reflect age at appendectomy, time since surgery, diagnostic misclassification, appendicitis-related immune processes, clinical selection, or residual confounding. Another recent Danish study also reported a protective association between appendectomy and IBD, although Scandinavian studies have highlighted that diagnostic misclassification may complicate interpretation, particularly when early IBD symptoms are initially diagnosed as appendicitis [23, 51]. Nevertheless, possible protection against IBD should not be interpreted as a clinical advantage of appendectomy, because IBD is relatively rare, the absolute risk reduction observed here was small, and IBD has a multifactorial aetiology involving genetic, immune, environmental, and microbiome-related factors.

There is no doubt that appendectomy can be life-saving in acute appendicitis. However, our present findings contribute to the broader discussion of whether non-operative management may be appropriate in selected uncomplicated or non-acute cases. The first appendectomy was performed in the 1880s [52, 53], and over the course of the 20^th^ century, the procedure became routine practice as anaesthetics, antibiotics, and laparoscopy reduced the risks [36]. At present, appendectomy is a common emergency procedure performed by gastrointestinal surgeons [33], both in the general population [34] and in children [36]—it is in fact the most common nonelective operation performed by surgeons [54]. Antibiotic treatment as an alternative to surgery in selected cases has been widely discussed [55], and our findings suggest that potential long-term disease associations should be considered alongside short-term risks and benefits when evaluating treatment strategies in children.

Related to these considerations, appendicitis prevalence varies substantially across populations and appears to be higher in industrialised settings than in many traditional or lower-income populations [56, 57]. More than half a century ago, this pattern was hypothesised to reflect differences in dietary fibre intake, particularly the lower fibre consumption associated with affluent Western diets [57–59]. More recent work suggests that this gradient may also reflect broader effects of urbanization, sanitation, enteric infection exposure, and diet-associated changes in gut microbiome composition and diversity [60–63]. Although our Danish registry data cannot directly test these ecological explanations, such variation underscores that both appendicitis risk and the long-term health associations of appendectomy may depend on environmental context.

The biological plausibility of long-term disease associations after childhood appendectomy is supported by the developmental timing of immune tissue function. The appendix and other ‘front-line’ immune tissues, from tonsils [28] to Peyer’s patches [64], are largest and have the most lymphoid nodules during childhood, after which they decrease in size with age. These tissues are located at major interfaces between the external environment and the immune system, including the nose, throat, and intestines, where they may contribute to early pathogen detection and immune education. Removal of such tissues during childhood may therefore plausibly influence immune development, microbial ecology, or host responses to later exposures. This interpretation is consistent with our observation that several disease associations extended beyond the digestive tract, including respiratory, allergic, infectious, and genitourinary outcomes.

The proposed microbial ‘safe house’ function of the appendix should not be interpreted as implying that the appendix is uniformly beneficial for adults. The same anatomical features that may help preserve commensal biofilms and support the re-inoculation of the gut after diarrheal disturbance could also, in some contexts, provide a niche for pathogenic, pathobiont, or dysbiotic microbial communities. Appendicitis itself may arise when obstruction, microbial overgrowth, immune activation, or local inflammatory processes disrupt normal appendix function. Thus, the appendix may have context-dependent effects, supporting host–microbiome resilience under some conditions while contributing to inflammation or pathogen persistence under others.

Our findings also have broader implications for how historically ‘vestigial’ immune-associated organs are viewed. A previous study showed that removal of tonsils and adenoids in childhood [65] was associated with altered long-term risk for several disease outcomes. Together with the present results, this suggests that removal of lymphoid tissues during childhood may have wider health associations than previously appreciated. The appendix is part of a broader immune network that includes the thymus, spleen, tonsils, adenoids, lymphatic vessels, and lymph nodes. Although our results cannot establish the mechanism, the breadth of disease associations observed here is consistent with the view that the appendix is not functionally redundant, particularly during childhood immune development.

These results are clinically relevant, biologically informative, and epidemiologically important. Clinically, they do not challenge appendectomy for acute appendicitis, but they do support continued evaluation of treatment alternatives for selected uncomplicated cases. Biologically, the broad pattern of associations is consistent with growing evidence that gut-associated immune tissue and the microbiome contribute to long-term health. Epidemiologically, this study illustrates how long-term registry data can identify disease associations that may otherwise be difficult to detect. More broadly, population differences in appendicitis prevalence, diarrheal disease burden, diet, sanitation, and microbiome composition highlight the need to study appendix function and appendectomy outcomes across diverse environmental contexts.

## LIMITATIONS

We were not able to assess disease risks beyond age 30 because follow-up was limited by the available registry period and the birth cohorts included. We therefore do not know whether the observed associations between appendectomy and disease risk persist, weaken, or strengthen later in adulthood. Our study was also restricted to Danish residents and may not generalize to populations with different healthcare systems, socioeconomic structures, environmental exposures, dietary patterns, enteric infection burdens, appendicitis prevalence, microbiome-related risk factors, or appendicitis management practices.

Although Danish registry data allowed adjustment for a broad range of parental, perinatal, socioeconomic, and demographic covariates, residual confounding remains possible. In particular, we could not fully distinguish associations attributable to appendectomy from those attributable to appendicitis itself, clinical decision-making, antibiotic exposure, underlying inflammatory susceptibility, infection vulnerability, or other shared factors that may influence both the likelihood of appendectomy and later disease risk. To reduce these possibilities, we excluded individuals with relevant disease diagnoses before age 12, assessed outcomes only after this age, adjusted for parental history of the same disease group, and tested for early general health differences between cases and controls in supplementary analyses. Nevertheless, these findings should be interpreted as long-term associations rather than definitive causal effects.

Despite these limitations, our study benefitted from comprehensive Danish registry linkage, longitudinal follow-up from birth, large sample size, broad disease outcome coverage, and detailed covariate adjustment that is difficult to achieve in smaller clinical or epidemiological cohorts.

## CONCLUSIONS

This study provides a broad population-based assessment of associations between childhood appendectomy and long-term disease risk. Appendectomy before age 12 was associated with increased risk of several digestive, respiratory, infectious, and genitourinary disease outcomes up to age 30, while IBD showed reduced RR. For common outcomes, modest RR increases translated into meaningful ARDs and low NNT-harm values because baseline disease prevalence was high. These findings suggest that the appendix may have lasting biological relevance, particularly during childhood immune development. They also support continued consideration of non-operative management for selected uncomplicated appendicitis cases, particularly in children whose immune systems are still developing.

## Supplementary Material

Supplementary_material_eoag011

## Data Availability

Data were made available by public authorities in accordance with The Danish Act on Processing of Personal Data (Act No. 429 of 31 May 2000) and deposited under terms of a contract at Statistics Denmark (https://www.dst.dk). They cannot leave the servers at Statistics Denmark.
