# Supplementary material for: Childhood appendectomy is linked with higher digestive, respiratory, and genitourinary disease risk but lower inflammatory bowel disease risk
Source: Evol Med Public Health. 2026 Jun 11;14(1):1–12. doi: 10.1093/emph/eoag011 (PMC13356811; doi:10.1093/emph/eoag011)
Supplement: Supplementary_material_eoag011 [file supplementary_material_eoag011.zip › Supplementary Methods.pdf]

Supplementary Methods

Study sample

Although registry data were available for some individuals older than 30 years, we restricted analyses to individuals born during 1979–1999 with follow-up through 2009 for two reasons. First, we required complete ascertainment of appendectomy status between birth and 12 years; for individuals born before 1979, appendectomy could have occurred prior to registry coverage and therefore could not be reliably classified. Second, for individuals born before 1979, information on pre-exposure disease history and key covariates was incomplete, which would introduce differential left-truncation and substantial missingness. Electronic health data were not available prior to 1977–1978 in Denmark, and post-2009 data were not available at the time permission for data access was granted.

Considerations related to propensity methods

Using Danish registry data with diagnoses recorded from birth, we matched cases and controls on pre-appendectomy health status (see "**Study sample from Danish registries**" in the main text and "**Testing for biases in early general health between cases and controls**" below). This approach addresses a core objective of propensity score methods: reducing baseline differences between exposed and unexposed individuals that could confound associations with subsequent outcomes.

In typical observational studies, complete pre-exposure disease histories are not available, so propensity score estimation relies on observed baseline covariates that act as proxies for underlying health status and risk. In contrast, because we observed diagnoses from birth, we were able to use direct indicators of pre-existing morbidity to assess and minimise baseline differences between cases and controls prior to surgery. This provides a strong basis for reducing confounding by measured pre-surgical health status, although—as with any non-randomised design—it cannot completely eliminate confounding from unmeasured factors.

A further practical consideration is that propensity score matching often reduces effective sample size. Given our aim to survey risks across a broad set of outcomes, and given that some outcomes were close to power thresholds even in this large dataset, approaches that substantially reduce sample size would limit feasibility for multi-outcome analyses. We therefore prioritised a design that retained maximal power while explicitly testing for baseline differences in early-life morbidity. Finally, propensity score approaches have limitations: they can adjust only for measured covariates, and their performance depends on model specification and the availability/quality of confounders.[1–3]

Power analyses

To reduce the risk of false-negative findings, we performed power analyses using the powerSurvEpi package in R to evaluate whether sample sizes were sufficient to test the null hypothesis of no association between appendectomy and subsequent disease incidence. The powerSurvEpi approach estimates the minimum sample size required to

detect a specified hazard ratio (or risk ratio under proportional hazards), given: (i) the estimated effect size, (ii) the variance of the exposure (appendectomy), (iii) the proportion diagnosed with the outcome, (iv) desired power, and (v) type I error rate. Most outcomes met these thresholds, including the 25 diseases examined. For analyses of "general health" (below), where relative risks were not pre-specified, we used a conservative postulated effect of $\exp(0.05) \approx 1.05$.

Converting RR to ARD and NNT

To aid interpretation of clinical and population impact,[4,5] we converted relative risks (RR) from Cox models and outcome prevalence in controls (control risk, CR) into absolute risk difference (ARD) and number needed to treat (NNT). We calculated:

- ARD (%) = 100 × CR × (RR − 1)
- NNT = 100 / |ARD|

Under this convention, positive ARD indicates increased risk (harm), and negative ARD indicates reduced risk (benefit). NNT is expressed as NNT-harm when ARD is positive and NNT-benefit when ARD is negative. Smaller absolute NNT values indicate larger effects (i.e., fewer individuals exposed for one additional outcome event).

Testing for biases in early general health between cases and controls

To assess whether appendectomy cases differed systematically from controls in morbidity prior to age 12 (i.e., whether cases were generally "sicker" before surgery), we tested two null hypotheses:

Null hypothesis 1: There is no difference between case and control age-at-diagnosis distributions for diagnoses occurring in the first 12 years of life (allowing multiple diagnoses per individual).
Null hypothesis 2: There is no difference between cases and controls in the distribution of age at first diagnosis within the first 12 years of life.

General health definition: To capture broad morbidity, we pooled ICD diagnosis groups spanning most major ICD-10 disease categories, using the same broad grouping framework described in Hollegaard et al.[6] This definition is intended to reflect overall morbidity burden rather than a single disease category.

Permutation procedure: For null hypothesis 1, we sampled from the control population (individuals without appendectomy during 1979–2009) a set of individuals equal in size to the appendectomy group. We compared the distributions of age at diagnosis for any general-health diagnosis within the first 12 years between cases and sampled controls using a Kolmogorov–Smirnov test. This procedure was repeated 1,000 times, each time sampling controls with replacement. The permutation p-value was computed as the proportion of permutations yielding a test result consistent with no distributional difference. In all permutations, $p > 0.05$, supporting the null hypothesis.

For null hypothesis 2, we repeated the same permutation procedure but considering only age at first general-health diagnosis. Again, all permutation p-values were > 0.05,

supporting the null hypothesis. Together, these results suggest that early-life general morbidity (up to age 12) did not differ materially between appendectomy cases and controls prior to surgery.

The Danish National Patient Registry (DNPR): coverage and data quality

Three features of the Danish healthcare system and DNPR support the completeness and quality of diagnostic ascertainment for both our outcome definitions and the pre-exposure “general health” comparisons:

1. **Universal access:** The Danish health care system is free for all residents so there are no financial concerns about GP or hospital visits, meaning there is minimal bias in health care access based on socioeconomic differences.
2. **Clinical recording incentives and standards:** Diagnoses are systematically recorded by clinicians as part of routine care and administrative reporting, supporting comprehensive documentation.
3. **Registry scope:** The DNPR captures inpatient and outpatient contacts and includes primary (and relevant secondary) diagnoses, along with dates and other encounter information, enabling longitudinal tracking of diagnoses and comorbidity.[7]

References

1. Garrido MM, Kelley AS, Paris J, et al. Methods for constructing and assessing propensity scores. Health services research. Oct 2014;49(5):1701-1720.
2. Shadish WR, Cook TD, Campbell DT. Experimental and quasi-experimental designs for generalized causal inference. Boston: Houghton Mifflin; 2001.
3. Pearl J. Causality : models, reasoning, and inference. 2nd ed. Cambridge ; New York: Cambridge University Press; 2009.
4. King NB, Harper S, Young ME. Use of relative and absolute effect measures in reporting health inequalities: structured review. BMJ. 2012;345:e5774.
5. Barratt A, Wyer PC, Hatala R, et al. Tips for learners of evidence-based medicine: 1. Relative risk reduction, absolute risk reduction and number needed to treat. CMAJ. Aug 17 2004;171(4):353-358.
6. Hollegaard B, Byars SG, Lykke J, Boomsma JJ. Parent-offspring conflict and the persistence of pregnancy-induced hypertension in modern humans. PLoS One. 2013;8(2):e56821.
7. Schmidt M, Schmidt SA, Sandegaard JL, Ehrenstein V, Pedersen L, Sorensen HT. The Danish National Patient Registry: a review of content, data quality, and research potential. Clinical epidemiology. 2015;7:449-490.
