# Supplementary material for: Childhood appendectomy is linked with higher digestive, respiratory, and genitourinary disease risk but lower inflammatory bowel disease risk
Source: Evol Med Public Health. 2026 Jun 11;14(1):1–12. doi: 10.1093/emph/eoag011 (PMC13356811; doi:10.1093/emph/eoag011)
Supplement: Supplementary_material_eoag011 [file supplementary_material_eoag011.zip › Table S1.pdf]

Table S1. Sample sizes used for each analysis including the number of individuals who underwent surgery between birth and 12 years, number diagnosed with a particular disease after 12 years and up to 30 years of age, and total sample size available. Sample sizes represent those after all exlcusions have been applied and are thus smaller than the sample in Table 1.

		appendectomy		
Disease		Surgery (*n*)	Disease (*n*)	Total (*n*)
**infectious**	**all**	8169	33968	968993
**allergic**	**all**	9461	16212	1051186
	**urticaria/angiodema**	9614	4264	1066826
**skin**	**all**	9229	47760	1037425
**respiratory**	**all**	7667	48135	888556
	**upper**	8616	31389	974249
	**lower**	8687	6669	988949
	**chronic lower**	9226	21203	1024866
	**asthma**	9267	20977	1028462
	**influenza**	9607	2515	1068065
	**pneumonia**	8949	6058	1016074
**digestive**	**all**	1150	35605	1017027
	**IBD**	9655	3855	1070898
	**liver**	9655	807	1070839
	**pancreatitis**	9655	623	1071086
	**ulcer**	9653	1481	1071026
**endocrine**	**all**	9439	24460	1053804
**genitourinary**	**all**	9338	21514	1053817
	**kidney infection**	9555	3370	1064795
**musculoskeletal**	**all**	9392	101036	1043348
**neoplasms**	**all**	9530	26849	1060330
	**benign**	9564	23229	1062520
**circulatory**	**all**	9612	11925	1067191
**nervous**	**all**	9585	7395	1065747
**mental**	**all**	9646	44875	1069698

Footnotes: Numbers are presented only for analyses with sufficient power for hypothesis testing (see methods)
