# Supplementary material for: Childhood appendectomy is linked with higher digestive, respiratory, and genitourinary disease risk but lower inflammatory bowel disease risk
Source: Evol Med Public Health. 2026 Jun 11;14(1):1–12. doi: 10.1093/emph/eoag011 (PMC13356811; doi:10.1093/emph/eoag011)
Supplement: Supplementary_material_eoag011 [file supplementary_material_eoag011.zip › Table S2.pdf]

Table S2. ICD-8 and ICD-10 classification codes used for A. main disease outcomes and B. covariates.

		**A. MAIN DISEASES**
**Infectious – all**	**ICD-8:**	00009,00019,00099,00199,00209,00219,00299,00300,00301,00308, 00309,00390,00391,00398,00399,00409,00419,00429,00439,00489, 00499,00509,00519,00529,00589,00599,00609,00690,00698,00699, 00709,00719,00720,00799,00809,00819,00820,00821,00829,00839, 00880,00889,00890,00899,00909,00919,00920,00921,00922,00928, 00929,00999,01099,01100,01101,01102,01103,01104,01105,01108, 01109,01200,01201,01208,01209,01210,01211,01218,01219,01229, 01239,01290,01291,01292,01299,01300,01301,01308,01309,01390, 01391,01399,01400,01401,01402,01409,01509,01519,01529,01589, 01599,01600,01601,01602,01603,01604,01605,01606,01608,01609, 01700,01701,01702,01708,01709,01710,01718,01719,01729,01739, 01790,01799,01809,01819,01899,01900,01901,01902,01903,01904, 01908,01909,01919,01920,01921,01929,01930,01931,01932,01938, 01939,01999,02009,02099,02199,02299,02309,02319,02329,02399, 02499,02599,02609,02700,02701,02708,02709,02719,02790,02799, 03009,03019,03029,03039,03099,03199,03299,03319,03399,03400, 03401,03409,03410,03411,03419,03599,03609,03610,03611,03612, 03618,03619,03689,03699,03799,03809,03819,03829,03880,03889, 03899,03909,03919,03990,03991,03992,03993,03999,04000,04009, 04199,04299,04399,04499,04509,04519,04599,04699,05009,05019, 05099,05199,05200,05201,05208,05209,05300,05301,05302,05303, 05308,05309,05400,05401,05402,05403,05404,05408,05409,05500, 05501,05508,05509,05600,05601,05608,05609,05709,05719,05789, 05799,06009,06099,06199,06209,06219,06239,06299,06309,06329, 06399,06499,06599,06600,06608,06609,06709,06749,06759,06799, 06809,06819,06829,06899,07000,07001,07002,07003,07004,07005, 07006,07007,07008,07009,07199,07200,07201,07202,07203,07204, 07208,07209,07399,07409,07419,07429,07499,07500,07501,07508, 07509,07699,07799,07809,07819,07829,07889,07899,07909,07910, 07911,07918,07919,07929,07939,07949,07951,07959,07982,07983, 07984,07989,07999,08099,08109,08119,08129,08199,08209,08219, 08229,08299,08309,08319,08389,08399,08409,08419,08429,08439, 08449,08459,08480,08489,08499,08509,08519,08599,08609,08699, 08709,08719,08789,08799,08809,08819,08899,08909,08999,10009, 10080,10089,10099,10199,10209,10219,10229,10269,10289,10299, 10309,10339,10399,10409,10499,11000,11001,11002,11003,11004, 11005,11008,11009,11109,11119,11129,11180,11189,11199,11200, 11201,11208,11209,11399,11499,11599,11609,11699,11709,11719, 11729,11739,11749,11789,11799,12009,12019,12029,12089,12099, 12109,12119,12129,12139,12199,12209,12219,12289,12299,12309, 12319,12329,12339,12349,12369,12399,12499,12509,12519,12529, 12539,12559,12589,12599,12609,12619,12689,12699,12709,12719, 12729,12739,12749,12759,12799,12809,12889,12899,12999,13100, 13101,13108,13109,13200,13201,13208,13209,13309,13399,13409, 13419,13429,13439,13449,13459,13469,13499,13599,13600,13601, 13602,13603,13609
	**ICD10:**	A00,A000,A001,A009,A01,A010,A011, A012,A013,A014,A02,A020,A021,A022,A022A,A022C,A022D, A022E,A028,A029,A03,A030,A031,A032,A033,A038,A039,

A04,A040,A041,A042,A043,A044,A045,A046,A047,A048,
A049,A05,A050,A051,A052,A053,A054,A058,A059,A06,
A060,A061,A062,A063,A063A,A064,A065,A066,A068,A068A,
A069,A07,A070,A071,A071A,A072,A073,A073A,A078,A079,
A08,A080,A081,A082,A083,A083A,A084,A085,A09,A099,
A15,A150,A150A,A150C,A150D,A151,A152,A153,A154,A154A,
A154B,A154C,A155,A155A,A156,A156A,A157,A158,A158A,A159,
A16,A160,A160A,A160B,A160C,A160D,A161,A162,A162A,A162B,
A162C,A162D,A163,A164,A164A,A164B,A164C,A164D,A165,A165A,
A167,A168,A168A,A168B,A168C,A169,A17,A170,A170A,A171,
A178,A178A,A178D,A178E,A179,A18,A180,A180A,A180B,A180C,
A180D,A180E,A180F,A180G,A181,A181B,A181C,A181D,A181F,A182,
A183,A183A,A183B,A183C,A183E,A184,A184A,A184C,A184E,A184G,
A184I,A185,A185D,A186,A187,A187A,A188,A188B,A188D,A188H,
A19,A190,A191,A192,A198,A199,A20,A209,A21,A219,
A229,A23,A239,A239A,A239B,A239C,A239D,A240,A240A,A244,
A25,A259,A269,A27,A270,A278,A278D,A279,A28,A280,
A281,A282,A282A,A282B,A288,A289,A30,A309,A31,A310,
A310A,A310C,A311,A311A,A311B,A318,A319,A320,A321,A321A,
A321B,A327,A328,A329,A339,A35,A359,A36,A360,A361,
A362,A363,A368,A368A,A368D,A369,A37,A370,A371,A378,
A379,A38,A389,A39,A390,A391,A392,A392A,A393,A394,
A395,A398,A398B,A399,A40,A400,A401,A402,A403,A408,
A409,A41,A410,A411,A411A,A412,A413,A414,A415,A418,
A419,A419A,A419B,A42,A420,A421,A422,A427,A428,A429,
A439,A449,A46,A469,A48,A480,A481,A482,A483,A484,
A488,A488A,A49,A490,A491,A491A,A492,A493,A498,A499,
A499A,A659,A669,A679,A68,A680,A681,A689,A69,A690,
A690A,A690C,A691,A691A,A691C,A691E,A691F,A691G,A692,A692A,
A692B,A692C,A692D,A692E,A692F,A692G,A698,A699,A70,A709,
A709A,A709B,A719,A74,A740,A748,A749,A75,A759,A77,
A779,A78,A789,A79,A799,A80,A800,A801,A802,A803,
A804,A809,A81,A810,A811,A812,A818,A819,A82,A829,
A830,A838,A839,A84,A840,A840A,A841,A848,A849,A85,
A850,A851,A851B,A852,A858,A86,A869,A87,A870,A871,
A872,A872A,A878,A879,A88,A880,A881,A888,A89,A899,
A90,A909,A91,A919,A929,A931,A94,A949,A959,A962,
A968,A969,A98,A984,A985,A985A,A985B,A985C,A988,A99,
A999,B00,B000,B000A,B001,B001A,B001B,B002,B002A,B002B,
B003,B004,B004A,B005,B005A,B005B,B005C,B005D,B005E,B005F,
B005G,B007,B008,B009,B01,B010,B011,B011A,B012,B018,
B019,B02,B020,B020A,B021,B022,B022A,B022B,B023,B023A,
B023B,B023C,B023D,B023E,B023F,B027,B028,B029,B03,B039,
B049,B05,B050,B051,B052,B053,B054,B058,B059,B06,
B060,B068,B069,B07,B079,B079A,B079B,B079C,B079D,B08,
B080,B080A,B080B,B081,B082,B083,B084,B084A,B085,B088,
B088A,B088C,B09,B099,B15,B150,B159,B159A,B16,B160,
B161,B162,B169,B17,B170,B171,B172,B178,B18,B180,
B181,B182,B188,B189,B19,B190,B199,B25,B250,B251,
B252,B253,B257,B258,B259,B26,B260,B261,B262,B263,
B268,B269,B27,B270,B271,B278,B279,B30,B300,B301,
B301A,B301B,B302,B303,B308,B309,B33,B330,B331,B332,
B333,B338,B34,B340,B341,B341A,B341B,B342,B343,B344,
B348,B349,B349A,B35,B350,B350A,B350C,B351,B352,B353,

		B354,B355,B356,B356A,B358,B358A,B359,B36,B360,B360A, B360B,B361,B362,B363,B368,B368A,B369,B37,B370,B370A, B371,B372,B372A,B372B,B373,B373A,B373B,B374,B374A,B375, B376,B377,B378,B378A,B378B,B378C,B379,B38,B389,B39, B399,B40,B409,B419,B429,B43,B439,B44,B440,B441, B441A,B441B,B442,B447,B448,B448A,B449,B450,B451,B451A, B452,B453,B457,B458,B459,B46,B469,B469A,B469C,B47, B470,B471,B479,B48,B480,B481,B482,B483,B484,B487, B488,B49,B499,B499A,B50,B509,B51,B519,B52,B529, B53,B530,B538,B54,B549,B55,B550,B550A,B551,B552, B559,B56,B560,B561,B569,B57,B579,B579A,B58,B580, B581,B582,B582A,B583,B588,B588A,B589,B59,B599,B600, B601,B601A,B601B,B602,B608,B649,B65,B650,B651,B652, B656,B658,B659,B66,B660,B661,B663,B664,B665,B668, B669,B67,B670,B671,B672,B673,B674,B675,B676,B677, B678,B679,B68,B680,B681,B689,B699,B700,B701,B71, B719,B729,B739,B74,B740,B749,B75,B759,B76,B760, B761,B768,B769,B769A,B77,B770,B778,B779,B78,B789, B79,B799,B80,B809,B81,B814,B818,B82,B820,B829, B83,B830,B838,B839,B85,B850,B851,B852,B853,B854, B86,B869,B869A,B869B,B87,B879,B88,B880,B881,B888, B888B,B888C,B888D,B889,B89,B899,B899A
**allergic – all**	**ICD-8:**	69299,69292,69291,69290,69289,69288,69287,69286,69285,69284, 69283,69282,69281,69280,69279,69269,69263,69262,69261,69260, 69259,69249,69243,69242,69241,69240,69239,69233,69232,69231, 69230,69229,69223,69222,69221,69220,69219,69213,69212,69211, 69210,69209,69203,69202,69201,69200,69109,69108,69101,69100, 50709,50708,50703,50702,50701,50700,36003,99949,70899,70891, 70890,70809
	**ICD10:**	T886,T805,T782A,T782,T780B,T780A,T780,L509,L508E,L508D, L508C,L508B,L508A,L508,L506A,L506,L505,L504,L503,L502B, L502A,L502,L501,L500,L50,L239A,L239,L238C,L238B,L238A, L238,L237A,L237,L236A,L236,L235I,L235H,L235G,L235F,L235E, L235D,L235C,L235B,L235A,L235,L234A,L234,L233A,L233,L232G, L232F,L232E,L232D,L232C,L232B,L232A,L232,L231A,L231,L230E, L230D,L230C,L230B,L230A,L230,L23,L209,L208D,L208C,L208B, L208A,L208,L200A,L200,L20,J304,J303A,J303,J302,J301C, J301B,J301A,J301,H101A,H101
**allergic – urticaria/angiodema**	**ICD-8:**	70809,70890,70891,70899
	**ICD10:**	L50,L500,L501,L502,L502A,L502B,L503,L504,L505,L506, L506A,L508,L508A,L508B,L508C,L508D,L508E,L509
**skin – all**	**ICD-8:**	68009,68019,68029,68039,68049,68059,68069,68080,68081,68089, 68099,68100,68101,68102,68103,68104,68105,68106,68107,68108, 68109,68200,68201,68202,68209,68219,68229,68239,68249,68259, 68290,68299,68399,68400,68408,68409,68500,68501,68509,68600, 68608,68609,68619,68690,68692,68693,68694,68695,68696,68699, 69000,69001,69002,69003,69008,69009,69300,69308,69309,69400, 69401,69402,69403,69405,69408,69409,69519,69529,69530,69538, 69539,69549,69590,69591,69599,69609,69610,69619,69620,69621, 69628,69629,69639,69649,69650,69659,69699,69709,69719,69790, 69799,69809,69819,69829,69830,69831,69839,69849,69890,69899, 70099,70100,70101,70108,70109,70110,70112,70114,70115,70118, 70119,70129,70139,70190,70191,70199,70200,70201,70202,70203,

	70209,70300,70301,70302,70303,70304,70305,70308,70309,70400,70401,70402,70403,70404,70405,70406,70408,70409,70509,70519,70590,70591,70592,70593,70599,70700,70701,70708,70709,70809,70890
**ICD10:**	L00,L009,L009A,L009B,L01,L010,L010A,L010B,L011,L02,L020,L020A,L020B,L020C,L021,L021A,L021B,L021C,L022,L022A,L022B,L022C,L022D,L022E,L022F,L022G,L022H,L022I,L022J,L022K,L022L,L022N,L022O,L022P,L022Q,L022R,L022S,L022T,L023,L023A,L023B,L023C,L023D,L024,L024A,L024B,L024C,L024D,L024E,L024F,L024G,L024H,L024I,L024J,L024K,L024L,L024M,L028,L028A,L028B,L028C,L029,L029A,L029B,L029C,L03,L030,L030A,L030B,L030C,L030D,L030E,L030F,L030G,L030H,L030I,L030J,L030K,L031,L031A,L031B,L031C,L031D,L031E,L031F,L031G,L031H,L031I,L032,L033,L033A,L033B,L033C,L033D,L033E,L033F,L038,L038A,L038B,L039,L039A,L04,L040,L040A,L040B,L040C,L041,L042,L042A,L043,L048,L049,L05,L050,L059,L08,L080,L080A,L081,L088,L088A,L088B,L088C,L089,L10,L100,L101,L102,L103,L104,L104A,L108,L108A,L109,L11,L110,L111,L118,L119,L12,L120,L121,L121A,L123,L128,L129,L13,L130,L131,L131A,L138,L138A,L138B,L138C,L139,L20,L200,L200A,L208,L208A,L208B,L208C,L208D,L209,L21,L210,L210A,L210B,L218,L218A,L218B,L218C,L218E,L218F,L218G,L219,L28,L280,L280A,L280B,L280C,L281,L282,L282A,L282B,L282C,L282E,L30,L300,L301,L301A,L301B,L302,L302A,L303,L304,L305,L305A,L308,L308A,L308B,L308C,L308D,L308E,L308F,L308G,L308H,L308I,L309,L40,L400,L400A,L400B,L400C,L401,L401A,L401B,L402,L402A,L402B,L403,L403A,L403B,L404,L405,L408,L408A,L408B,L408C,L408D,L408E,L409,L41,L410,L410A,L410B,L410C,L411,L412,L413,L414,L415,L418,L419,L42,L429,L43,L430,L431,L433,L438,L438A,L438B,L438C,L438D,L438E,L439,L44,L440,L441,L442,L443,L448,L448A,L448B,L448C,L448D,L449,L50,L500,L501,L502,L502A,L502B,L503,L504,L505,L506,L506A,L508,L508A,L508B,L508C,L508D,L508E,L509,L51,L510,L511,L511A,L512,L518,L518A,L519,L52,L529,L53,L531,L532,L533,L538,L538A,L538B,L538C,L538D,L539,L539A,L539B,L60,L600,L601,L602,L603,L603A,L603B,L604,L605,L608,L608A,L608C,L608D,L608E,L608F,L608G,L608H,L608I,L609,L63,L630,L631,L632,L638,L638A,L639,L64,L648,L649,L65,L650,L651,L652,L658,L658A,L658B,L658C,L659,L66,L660,L661,L662,L663,L664,L668,L668A,L669,L67,L670,L671,L671D,L671E,L678,L678A,L678B,L678D,L679,L68,L682,L683,L688,L689,L71,L711,L718,L718A,L718B,L718C,L718D,L718E,L719,L72,L720,L721,L721A,L721B,L722,L728,L729,L73,L730,L731,L732,L738,L738A,L738B,L738D,L738E,L738F,L738H,L738I,L739,L74,L740,L741,L742,L743,L744,L744A,L748,L748A,L749,L75,L750,L751,L752,L752A,L758,L759,L84,L849,L849A,L849B,L87,L870,L871,L872,L878,L878A,L879,L88,L889,L889B,L889D,L89,L899,L899A,L899B,L899C,L899D,L899E,L899F,L90,L900,L900A,L900B,L901,L902,L903,L904,L904A,L905,L905A,L905B,L905C,L905D,L906,L906A,L906B,L906C,L906D,L908,L908A,L908B,L908D,L908F,L908G,L908J,L908K,L909,L91,L910,L910A,L918,L918B,L919,L92,L920,L920A,L920C,L921,L922,L923,L923A,L923B,L928,L928A,L928D,L928E,L928G,L929,L929A,L929B,L94,L940,L940A,L940B,L941,L942,L942A,L942B,L943,

		L944,L945,L946,L948,L949,L95,L950,L950A,L951,L958, L958A,L958B,L959,L97,L979,L979A,L979B,L979C,L979D,L979E, L98,L980,L981,L981A,L982,L983,L984,L984A,L984B,L984C, L984D,L984E,L985,L985A,L985B,L985C,L985D,L985E,L985F,L985G, L985H,L985I,L986,L988,L988A,L988B,L988C,L988D,L989
**respiratory – all**	**ICD-8:**	46099,46100,46101,46102,46103,46104,46108,46109,46200,46201, 46208,46209,46300,46301,46302,46308,46309,46400,46401,46402, 46403,46408,46409,46599,46600,46601,46602,46608,46609,47099, 47100,47101,47108,47109,47200,47201,47209,47300,47308,47309, 47400,47408,47409,48099,48100,48101,48108,48109,48209,48219, 48229,48239,48290,48299,48300,48308,48309,48499,48599,48699, 49000,49001,49009,49100,49101,49102,49103,49104,49108,49109, 49200,49201,49208,49209,49300,49301,49302,49308,49309,50000, 50001,50002,50008,50009,50199,50209,50210,50211,50212,50213, 50214,50215,50218,50219,50300,50301,50302,50303,50304,50305, 50306,50307,50308,50309,50400,50499,50500,50501,50502,50503, 50504,50508,50509,50699,50700,50701,50702,50703,50708,50709, 50800,50801,50802,50803,50804,50805,50806,50807,50809,51000, 51001,51002,51003,51004,51008,51009,51100,51101,51108,51109, 51119,51120,51121,51122,51129,51299,51300,51301,51302,51309, 51400,51408,51409,51700,51701,51709,51899,51909,51919,51920, 51921,51922,51923,51929,51990,51991,51992,51993,51999
	**ICD10:**	J00,J009,J009A,J009B,J009C,J01,J010,J010A,J010B,J011, J011A,J011B,J012,J012A,J012B,J013,J013B,J014,J014A,J018, J019,J02,J020,J020A,J028,J029,J029A,J029B,J029C,J03, J030,J038,J039,J039A,J039B,J039C,J039D,J039E,J039F,J04, J040,J040A,J040B,J040D,J041,J042,J042A,J05,J050,J051, J06,J060,J068,J069,J09,J091,J091A,J091B,J099,J10, J100,J101,J101A,J101B,J101C,J108,J108A,J108B,J108C,J11, J110,J111,J111A,J111B,J111C,J118,J118A,J118B,J118C,J12, J120,J121,J122,J128,J129,J13,J139,J139A,J139B,J14, J149,J149A,J149B,J15,J150,J151,J152,J153,J154,J155, J156,J156A,J157,J158,J159,J16,J160,J168,J18,J180, J181,J182,J188,J189,J20,J200,J201,J202,J203,J204, J205,J206,J207,J208,J209,J209A,J21,J210,J218,J219, J22,J229,J30,J300,J300A,J300B,J301,J301A,J301B,J301C, J302,J303,J303A,J304,J31,J310,J310A,J310B,J310D,J310E, J310F,J311,J312,J312A,J312B,J32,J320,J321,J322,J323, J324,J328,J329,J33,J330,J330A,J330B,J331,J331A,J338, J338A,J338B,J338C,J338D,J339,J34,J340,J340A,J340B,J340C, J340D,J340E,J340F,J340J,J341,J341A,J341B,J342,J343,J343A, J348,J348A,J348B,J348C,J35,J350,J351,J351A,J351B,J352, J353,J358,J358A,J358C,J359,J36,J369,J37,J370,J370A, J370B,J370C,J371,J38,J380,J380A,J380B,J381,J381A,J381B, J382,J382A,J383,J383A,J383B,J383C,J383D,J383E,J384,J384A, J384B,J384C,J384D,J385,J385A,J385B,J386,J387,J387A,J387B, J387D,J387E,J387G,J39,J390,J390A,J390B,J390C,J391,J391A, J392,J392A,J392B,J392C,J392D,J398,J399,J40,J409,J41, J410,J411,J418,J42,J429,J429A,J429B,J43,J430,J430A, J431,J431A,J432,J438,J439,J439A,J44,J440,J441,J448, J448A,J448B,J449,J45,J450,J450A,J451,J451A,J458,J459, J459A,J46,J469,J47,J479,J80,J801,J809,J81,J819, J82,J821,J829,J84,J840,J840A,J840B,J841,J841A,J841B, J841C,J841D,J841E,J842,J843,J848,J848A,J849,J85,J850,

		J850A,J851,J852,J853,J86,J860,J860A,J869,J869A,J90, J909,J92,J929,J93,J930,J931,J938,J939,J94,J940, J941,J942,J942A,J948,J948A,J949,J96,J960,J961,J969, J98,J980,J980A,J980B,J980C,J980D,J980E,J980G,J980H,J980I, J981,J982,J982A,J983,J984,J984B,J984C,J985,J985A,J985B, J985C,J985D,J986,J986A,J986B,J986C,J986D,J988,J989
**respiratory – upper**	**ICD-8:**	46099,46100,46101,46102,46103,46104,46108,46109,46200,46201, 46208,46209,46300,46301,46302,46308,46309,46400,46401,46402, 46403,46408,46409,46599,50000,50001,50002,50008,50009,50199, 50209,50210,50211,50212,50213,50214,50215,50218,50219,50300, 50301,50302,50303,50304,50305,50306,50307,50308,50309,50400, 50499,50500,50501,50502,50503,50504,50508,50509,50699,50700, 50701,50702,50703,50708,50709,50800,50801,50802,50803,50804, 50805,50806,50807,50809
	**ICD10:**	J00,J009,J009A,J009B,J009C,J01,J010,J010A,J010B,J011, J011A,J011B,J012,J012A,J012B,J013,J013B,J014,J014A,J018, J019,J02,J020,J020A,J028,J029,J029A,J029B,J029C,J03, J030,J038,J039,J039A,J039B,J039C,J039D,J039E,J039F,J04, J040,J040A,J040B,J040D,J041,J042,J042A,J05,J050,J051, J06,J060,J068,J069,J30,J300,J300A,J300B,J301,J301A, J301B,J301C,J302,J303,J303A,J304,J31,J310,J310A,J310B, J310D,J310E,J310F,J311,J312,J312A,J312B,J32,J320,J321, J322,J323,J324,J328,J329,J33,J330,J330A,J330B,J331, J331A,J338,J338A,J338B,J338C,J338D,J339,J34,J340,J340A, J340B,J340C,J340D,J340E,J340F,J340J,J341,J341A,J341B,J342, J343,J343A,J348,J348A,J348B,J348C,J35,J350,J351,J351A, J351B,J352,J353,J358,J358A,J358C,J359,J36,J369,J37, J370,J370A,J370B,J370C,J371,J38,J380,J380A,J380B,J381, J381A,J381B,J382,J382A,J383,J383A,J383B,J383C,J383D,J383E, J384,J384A,J384B,J384C,J384D,J385,J385A,J385B,J386,J387, J387A,J387B,J387D,J387E,J387G,J39,J390,J390A,J390B,J390C, J391,J391A,J392,J392A,J392B,J392C,J392D,J398,J399
**respiratory – lower**	**ICD-8:**	46600,46601,46602,46608,46609,48099,48100,48101,48108,48109, 48209,48219,48229,48239,48290,48299,48300,48308,48309,48499, 48599,48699
	**ICD10:**	J12,J120,J121,J122,J128,J129,J13,J139,J139A,J139B, J14,J149,J149A,J149B,J15,J150,J151,J152,J153,J154, J155,J156,J156A,J157,J158,J159,J16,J160,J168,J18, J180,J181,J182,J188,J189,J20,J200,J201,J202,J203, J204,J205,J206,J207,J208,J209,J209A,J21,J210,J218, J219,J22,J229
**respiratory – lower-chronic**	**ICD-8:**	49000,49001,49009,49100,49101,49102,49103,49104,49108,49109, 49200,49201,49208,49209,49300,49301,49302,49308,49309
	**ICD10:**	J40,J409,J41,J410,J411,J418,J42,J429,J429A,J429B, J43,J430,J430A,J431,J431A,J432,J438,J439,J439A,J44, J440,J441,J448,J448A,J448B,J449,J45,J450,J450A,J451, J451A,J458,J459,J459A,J46,J469,J47,J479
**respiratory – asthma**	**ICD-8:**	49300,49301,49302,49308,49309
	**ICD10:**	J45,J450,J450A,J451,J451A,J458,J459,J459A,J46,J469
**respiratory – influenza**	**ICD-8:**	47099,47100,47101,47108,47109,47200,47201,47209,47300,47308 47309,47400,47408,47409
	**ICD10:**	J09,J091,J091A,J091B,J099,J10,J100,J101,J101A,J101B, J101C,J108,J108A,J108B,J108C,J11,J110,J111,J111A,J111B,

		J111C,J118,J118A,J118B,J118C
**respiratory – pneumonia**	**ICD-8:**	48099,48100,48101,48108,48109,48209,48219,48229,48239,48290, 48299,48300,48308,48309,48499,48599,48699
	**ICD10:**	J12,J120,J121,J122,J128,J129,J13,J139,J139A,J139B, J14,J149,J149A,J149B,J15,J150,J151,J152,J153,J154, J155,J156,J156A,J157,J158,J159,J16,J160,J168,J18, J180,J181,J182,J188,J189
**digestive – all**	**ICD-8:**	53000,53001,53002,53008,53009,53090,53091,53092,53093,53094, 53095,53096,53097,53098,53099,53100,53101,53108,53109,53190, 53191,53192,53193,53194,53195,53196,53198,53199,53209,53290, 53291,53299,53309,53390,53391,53399,53409,53490,53491,53499, 53500,53501,53502,53503,53504,53505,53506,53508,53509,53600, 53608,53609,53619,53690,53691,53692,53693,53699,53700,53701, 53702,53703,53704,53705,53706,53709,54000,54001,54002,54008, 54009,54090,54091,54092,54098,54099,54199,54200,54209,54300, 54301,54302,54309,55000,55001,55008,55009,55100,55101,55109, 55119,55129,55130,55131,55139,55180,55181,55182,55183,55189, 55199,55299,55309,55319,55329,55339,55380,55381,55389,55399, 56000,56001,56002,56008,56009,56019,56020,56021,56022,56028, 56029,56030,56038,56039,56090,56091,56092,56093,56094,56099, 56100,56101,56102,56103,56108,56109,56200,56208,56209,56210, 56211,56212,56218,56219,56300,56301,56302,56308,56309,56319, 56399,56419,56490,56499,56700,56701,56702,56703,56704,56708, 56709,56899,56900,56901,56904,56905,56906,56907,56908,56912, 56913,56914,56915,56916,56917,56920,56999,57000,57001,57002, 57008,57009,57109,57111,57119,57190,57191,57192,57193,57194, 57199,57200,57201,57209,57300,57301,57302,57303,57304,57305, 57309,57400,57401,57402,57403,57404,57405,57406,57407,57408, 57409,57500,57501,57502,57503,57504,57508,57509,57600,57601, 57602,57603,57604,57605,57609,57700,57701,57702,57703,57704, 57708,57709,57711,57719,57790,57791,57792,57793,57799
	**ICD10:**	K20,K209,K209A,K209B,K209C,K21,K210,K210A,K219,K219A, K22,K220,K220A,K221,K221A,K221B,K221C,K221D,K221E,K222, K222A,K222B,K222C,K225,K225A,K225B,K228,K228A,K228B,K228C, K228D,K228E,K228F,K228G,K228H,K228I,K229,K25,K250,K250A, K250B,K250C,K250D,K250E,K250F,K251,K251A,K251B,K251C,K251D, K251E,K252,K252A,K252B,K252C,K252D,K252E,K253,K253A,K253B, K253C,K253D,K253E,K254,K254A,K254B,K254C,K254D,K254E,K254F, K254G,K254H,K254I,K254J,K255,K255A,K255D,K255E,K255F,K255H, K255I,K255J,K256,K256B,K256D,K256E,K256F,K256H,K256I,K256J, K257,K257A,K257B,K257C,K257D,K257E,K259,K259A,K26,K260, K260A,K261,K261A,K262,K262A,K263,K263A,K264,K264A,K264B, K264C,K264D,K265,K265A,K265B,K265D,K266,K266A,K266B,K266C, K266D,K267,K267A,K267B,K269,K27,K270,K271,K272,K273, K274,K275,K276,K277,K279,K28,K280,K280A,K280B,K281, K281A,K281B,K282,K282B,K283,K283A,K283B,K283C,K284,K284A, K284B,K284C,K284D,K284E,K284F,K285,K285A,K285B,K285C,K285D, K285F,K286,K286A,K286B,K287,K287A,K287B,K287C,K289,K29, K290,K291,K293,K294,K295,K296,K296A,K296B,K296C,K297, K298,K298A,K299,K30,K309,K31,K310,K310A,K311,K311A, K312,K312A,K312B,K312C,K313,K314,K314A,K315,K315A,K315B, K316,K316A,K316B,K316C,K316D,K316E,K318,K318A,K318B,K318C, K318E,K318F,K318G,K318H,K319,K35,K350,K350A,K351,K351A,

		K359,K359A,K359B,K36,K369,K37,K379,K38,K380,K381, K381A,K382,K383,K388,K388A,K388B,K388C,K388D,K389,K40, K400,K401,K402,K402A,K403,K403A,K403B,K404,K409,K41, K410,K411,K412,K412A,K413,K413A,K413B,K414,K419,K42, K420,K420A,K420B,K421,K429,K43,K430,K430A,K430B,K431, K439,K439A,K439A1,K439A2,K439A3,K439A4,K44,K440,K440A,K440B, K441,K449,K45,K450,K450A,K450B,K450C,K450F,K450I,K451, K451A,K451C,K451E,K451F,K451H,K451I,K458,K458A,K458B,K458C, K458F,K458G,K458H,K458I,K458J,K458K,K458M,K458P,K46,K460, K460A,K460B,K461,K469,K51,K510,K511,K512,K512A,K513, K513A,K514,K515,K518,K518A,K518B,K519,K55,K550,K550A, K550B,K550C,K550D,K550E,K550F,K550G,K550H,K551,K551A,K551B, K551C,K551D,K551E,K551F,K552,K553,K553A,K553B,K553C,K558, K559,K56,K560,K560A,K560B,K561,K561A,K561B,K561C,K561D, K562,K562A,K562B,K562C,K563,K564,K564A,K564B,K565,K565A, K565B,K565C,K565D,K565E,K566,K566A,K566B,K566C,K566D,K566E, K566F,K566G,K566H,K567,K57,K570,K570A,K570B,K570C,K571, K571A,K571B,K572,K572A,K572B,K572C,K573,K573A,K573B,K573C, K573D,K573E,K573F,K574,K574A,K575,K578,K579,K579A,K58, K580,K580A,K589,K589A,K63,K630,K631,K631A,K632,K632A, K632B,K632C,K632D,K632E,K632F,K632G,K632H,K632I,K632J,K633, K634,K638,K638A,K638B,K638C,K638D,K638E,K638F,K639,K65, K650,K650A,K650B,K650C,K650D,K650E,K650F,K650G,K650H,K650I, K650J,K650K,K650L,K650M,K650N,K650O,K650P,K658,K658A,K658B, K658C,K658D,K658E,K658F,K658G,K658I,K659,K66,K660,K660A, K660B,K660C,K660D,K660E,K660F,K661,K668,K668A,K668B,K669, K72,K720,K720A,K720C,K720D,K720E,K720F,K721,K729,K73, K730,K731,K732,K732A,K732B,K732C,K732D,K732E,K732F,K732G, K738,K739,K74,K740,K740A,K740B,K741,K742,K743,K743A, K744,K745,K746,K746A,K746B,K746C,K746D,K746E,K746F,K746G, K75,K750,K750A,K750B,K750C,K750D,K751,K751A,K752,K753, K758,K759,K76,K760,K760A,K760B,K760C,K761,K761A,K762, K763,K764,K764A,K765,K766,K766A,K766B,K767,K768,K768A, K768B,K768C,K768E,K769,K80,K800,K800A,K800B,K800C,K800D, K801,K801A,K801B,K801C,K801D,K802,K802A,K802B,K802C,K802D, K802E,K802F,K803,K803A,K803B,K803C,K804,K804A,K804B,K804C, K804D,K804E,K805,K805A,K805B,K805C,K805D,K805E,K805F,K805G, K805H,K805I,K805J,K805K,K808,K81,K810,K810A,K810B,K810C, K810D,K811,K818,K819,K82,K820,K820A,K821,K822,K822A, K823,K823A,K823B,K824,K828,K828B,K828D,K828F,K828G,K828H, K828I,K828J,K828K,K829,K83,K830,K830A,K830B,K830C,K830D, K830E,K830F,K830G,K831,K831A,K831B,K832,K832A,K833,K833A, K833B,K834,K835,K838,K838B,K838D,K838E,K839,K85,K859, K859A,K859B,K859C,K859D,K859E,K859F,K86,K861,K861A,K861B, K861C,K861D,K862,K863,K868,K868A,K868B,K868C,K868D,K868E, K868F,K868G,K868I,K868L,K868M,K868N,K869
**Digestive – inflammatory bowel disease (excl. Crohn's disease)**	**ICD8**	56301,56319
	**ICD10**	K51,K510,K511,K512,K512A,K513,K513A,K514,K515,K518,K518A, K518B,K519
**Digestive – liver disease (excluding liver disease due to alcohol/drugs)**	**ICD8**	57000,57001,57002,57008,57009,57119,57190,57191,57192,57193, 57194,57199,57200,57201,57209,57300,57301,57302,57303,57304, 57305,57309
	**ICD10**	K72,K720,K720A,K720C,K720D,K720E,K720F,K721,K729,K73,K730,

		K730,K731,K732,K732A,K732B,K732C,K732D,K732E,K732F,K732G, K738,K739,K74,K740,K740A,K740B,K741,K742,K743,K743A,K744, K745,K746,K746A,K746B,K746C,K746D,K746E,K746F,K746G,K75, K750,K750A,K750B,K750C,K750D,K751,K751A,K752,K753,K758, K759,K76,K760,K760A,K760B,K760C,K761,K761A,K762,K763,K764, K764A,K765,K766,K766A,K766B,K767,K768,K768A,K768B,K768C, K768E,K769
**Digestive – pancreatitis**	**ICD8**	57700,57701,57702,57703,57704,57708,57709
	**ICD10**	K85,K859,K859A,K859B,K859C,K859D,K859E,K859F
**Digestive – ulcer disease**	**ICD8**	53091,53098,53100,53101,53108,53109,53190,53191,53192,53193, 53194,53195,53196,53198,53199,53209,53290,53291,53299,53309, 53390,53391,53399,53409,53490,53491,53499
	**ICD10**	K221,K221A,K221B,K221C,K221D,K221E,K25,K250,K250A,K250B, K250C,K250D,K250E,K250F,K251,K251A,K251B,K251C,K251D,K251E, K252,K252A,K252B,K252C,K252D,K252E,K253,K253A,K253B,K253C, K253D,K253E,K254,K254A,K254B,K254C,K254D,K254E,K254F,K254G, K254H,K254I,K254J,K255,K255A,K255D,K255E,K255F,K255H,K255I, K255J,K256,K256B,K256D,K256E,K256F,K256H,K256I,K256J,K257, K257A,K257B,K257C,K257D,K257E,K259,K259A,K26,K260,K260,K260A, K261,K261A,K262,K262A,K263,K263A,K264,K264A,K264B,K264C,K264D, K265,K265A,K265B,K265D,K266,K266A,K266B,K266C,K266D,K267, K267A,K267B,K269,K27,K270,K271,K272,K273,K274,K275,K276,K277, K279,K28,K280,K280A,K280B,K281,K281A,K281B,K282,K282B,K283, K283A,K283B,K283C,K284,K284A,K284B,K284C,K284D,K284E,K284F, K285,K285A,K285B,K285C,K285D,K285F,K286,K286A,K286B,K287,K287A, K287B,K287C,K289
**endocrine – all**	**ICD-8:**	24009,24019,24099,24109,24119,24199,24200,24201,24208,24209, 24219,24220,24228,24229,24400,24401,24402,24403,24408,24409, 24500,24501,24502,24503,24504,24508,24509,24600,24609,25000, 25001,25002,25003,25004,25005,25006,25007,25008,25009,25100, 25101,25102,25103,25108,25109,25200,25201,25202,25203,25204, 25205,25208,25209,25210,25211,25218,25219,25299,25300,25301, 25302,25308,25309,25310,25311,25312,25313,25314,25315,25318, 25319,25329,25390,25399,25499,25500,25501,25508,25509,25510, 25511,25512,25518,25519,25529,25590,25599,25800,25801,25809, 25819,25890,25891,25892,25899,26009,26010,26019,26089,26099, 26199,26299,26309,26319,26380,26381,26389,26399,26401,26408, 26409,26509,26519,26529,26599,26609,26680,26689,26699,26700, 26708,26709,26899,26900,26901,26902,26909,26919,26990,26991, 26992,26999,27400,27401,27408,27409,27509,27519,27529,27539, 27549,27559,27590,27599,27600,27601,27602,27608,27609,27799, 27809,27819,27829,27899,27900,27901,27902,27903,27904,27905, 27906,27907,27908,27909
	**ICD10:**	E01,E010,E010A,E011,E011A,E012,E018,E02,E029,E03, E033,E034,E035,E038,E039,E04,E040,E041,E041A,E042, E042A,E048,E048A,E049,E05,E050,E050A,E050B,E050C,E050D, E051,E052,E053,E054,E055,E058,E058A,E058B,E059,E059A, E06,E060,E060A,E060B,E061,E061A,E061B,E061C,E062,E063, E063A,E063B,E063C,E065,E065A,E065C,E069,E07,E070,E070A, E078,E078A,E078B,E078E,E079,E11,E110,E110A,E110B,E110C, E110D,E110E,E111,E112,E113,E114,E115,E115A,E115B,E115C, E115D,E116,E117,E118,E119,E119A,E13,E130,E131,E132, E133,E134,E135,E136,E137,E138,E139,E14,E140,E140A,

		E140B,E140C,E140D,E141,E142,E143,E144,E145,E145A,E145B, E145C,E145D,E146,E147,E148,E149,E16,E161,E161A,E161B, E161C,E161D,E162,E163,E168,E168A,E168C,E168E,E169,E20, E200,E201,E208,E209,E21,E210,E210A,E210B,E211,E212, E213,E213A,E213B,E214,E215,E22,E220,E220A,E220C,E221, E222,E229,E23,E230,E230A,E230B,E230C,E230D,E230E,E230F, E230G,E230H,E230I,E232,E233,E236,E236A,E236B,E236C,E236E, E237,E24,E240,E241,E243,E248,E249,E26,E260,E260A, E260B,E261,E268,E268A,E269,E27,E271,E271A,E272,E272A, E274,E274A,E274B,E274C,E274D,E274E,E275,E275A,E278,E279, E31,E310,E310A,E311,E318,E319,E32,E320,E321,E328, E329,E34,E340,E341,E342,E349,E40,E409,E41,E419, E42,E429,E43,E439,E439A,E44,E440,E441,E45,E459, E46,E469,E47,E470,E50,E500,E501,E502,E503,E504, E505,E506,E507,E507A,E508,E508A,E509,E51,E511,E512, E513,E519,E52,E529,E529A,E53,E530,E531,E538,E538A, E538B,E538C,E538D,E538E,E539,E549,E55,E559,E56,E560, E561,E568,E569,E58,E589,E59,E599,E599A,E60,E609, E609A,E609B,E609C,E609D,E61,E610,E611,E612,E613,E614, E615,E616,E617,E618,E619,E63,E630,E631,E638,E639, E64,E640,E641,E642,E643,E648,E648A,E649,E649A,E649B, E65,E658,E658A,E658B,E659,E66,E660,E660A,E660B,E660C, E660D,E660E,E660F,E660G,E660H,E662,E662A,E668,E669,E67, E670,E671,E672,E673,E678,E68,E689,E78,E780,E780A, E780C,E780D,E780E,E781,E781A,E781B,E781C,E781D,E781E,E782, E782A,E782B,E782C,E782D,E782E,E782F,E782G,E782H,E782I,E782J, E783,E783A,E785
**genitourinary – all**	**ICD-8:**	58000,58001,58002,58008,58009,58199,58200,58201,58202,58208, 58209,58300,58301,58302,58308,58309,58499,59009,59010,59011, 59012,59013,59014,59015,59019,59020,59021,59029,59099,59100, 59101,59108,59109,59200,59201,59202,59203,59204,59205,59208, 59209,59309,59319,59320,59321,59322,59323,59324,59325,59326, 59327,59329,59330,59331,59338,59339,59340,59341,59349,59350, 59351,59352,59359,59400,59401,59409,59500,59501,59502,59503, 59504,59508,59509,59600,59601,59602,59603,59604,59605,59606, 59607,59608,59609,59700,59701,59702,59703,59708,59709,59800, 59801,59802,59803,59808,59809,59900,59901,59902,59903,59904, 59905,59906,59909,60100,60101,60102,60103,60108,60109,61200, 61201,61202,61203,61204,61208,61209,61300,61301,61302,61303, 61304,61308,61309,61499,61600,61601,61602,61603,61608,61609, 61619,61629,61690,61699,62009,62090,62099
	**ICD10:**	N00,N000,N001,N002,N003,N004,N005,N005A,N005C,N006, N007,N008,N008A,N008B,N009,N01,N010,N011,N012,N013, N014,N015,N015C,N016,N017,N018,N018A,N018B,N019,N03, N030,N031,N032,N033,N034,N035,N035A,N035C,N036,N037, N038,N038A,N038B,N039,N05,N050,N051,N052,N053,N054, N055,N055B,N055C,N056,N057,N058,N058A,N058B,N059,N06, N060,N061,N062,N063,N064,N065,N065C,N066,N067,N068, N068A,N068B,N069,N10,N109,N11,N110,N110A,N111,N111A, N111B,N112,N118,N119,N12,N129,N13,N130,N131,N131A, N132,N132A,N132B,N133,N133A,N134,N135,N135A,N135B,N135C, N136,N136A,N136B,N136C,N136D,N137,N138,N138A,N139,N139A, N139B,N15,N150,N151,N151A,N151B,N158,N159,N17,N170, N170A,N171,N171A,N172,N172B,N172C,N178,N179,N18,N180,

		N188,N188A,N188B,N189,N19,N199,N20,N200,N200A,N200F, N200I,N200M,N200S,N200X,N200X1,N200X2,N200Y,N200Z,N201,N201A, N201F,N201I,N201M,N201S,N201X,N201Y,N201Z,N202,N202A,N202F, N202I,N202M,N202S,N202X,N202X2,N202Y,N202Z,N209,N209A,N21, N210,N210A,N211,N218,N219,N23,N239,N25,N250,N250B, N250C,N250D,N251,N258,N258A,N258B,N258C,N259,N26,N269, N27,N270,N271,N279,N28,N280,N280A,N280B,N280D,N281, N288,N288A,N288B,N288D,N288E,N288F,N288G,N288H,N289,N289A, N30,N300,N301,N302,N303,N303A,N308,N308A,N308B,N308C, N308D,N308E,N308F,N308G,N308H,N308J,N308K,N309,N31,N310, N311,N312,N318,N318A,N319,N319B,N32,N320,N320A,N320B, N321,N321A,N322,N323,N324,N325,N325A,N325B,N328,N328A, N328B,N328C,N328D,N328E,N329,N34,N340,N340A,N340B,N340C, N341,N342,N342A,N342B,N342C,N342D,N342E,N342F,N342G,N343, N35,N351,N358,N358A,N359,N36,N360,N360A,N360B,N360C, N361,N362,N363,N368,N368A,N368C,N368D,N368E,N369,N39, N391,N392,N394,N394A,N394B,N394C,N398,N399,N41,N410, N411,N412,N412A,N413,N413A,N418,N419,N679,N70,N700, N700A,N700B,N700C,N700D,N700E,N700F,N700G,N701,N701A,N701B, N701C,N701D,N701E,N701F,N701G,N701H,N702,N709,N709B,N709C, N71,N710,N710A,N710B,N710C,N710D,N710E,N710F,N711,N711A, N711B,N711C,N711D,N711F,N719,N72,N729,N729A,N729B,N729C, N729D,N729E,N729F,N73,N730,N730B,N730C,N730E,N731,N731B, N731C,N732,N732A,N732B,N732C,N732E,N733,N733A,N734,N734A, N735,N735A,N736,N736A,N736B,N738,N738A,N738B,N738C,N739
**genitourinary – kidney infection***	**ICD-8:**	59009,59010,59011,59012,59013,59014,59015, 59019,59020,59021,59029,59099
	**ICD10:**	N10,N109,N11,N110,N110A,N111,N111A,N111B,N112,N118, N119,N12,N129

**** does not include urinary tract infections (N39.0, 599.0)***

**musculoskeletal – all**	**ICD-8:**	71000,71001,71002,71003,71004,71008,71009,71199,71219,71229, 71239,71249,71259,71300,71301,71302,71303,71304,71305,71306, 71308,71309,71310,71311,71312,71313,71314,71315,71318,71319, 71490,71491,71492,71493,71494,71499,71599,71609,71619,71709, 71719,71729,71790,71791,71799,71899,72000,72001,72002,72003, 72008,72009,72010,72011,72012,72013,72018,72019,72029,72030, 72031,72032,72038,72039,72199,72220,72221,72222,72223,72224, 72225,72289,72299,72309,72319,72390,72391,72392,72393,72394, 72395,72399,72409,72419,72499,72500,72501,72509,72510,72511, 72519,72589,72599,72699,72709,72719,72729,72739,72749,72759, 72769,72779,72789,72799,72809,72819,72829,72839,72849,72859, 72869,72879,72889,72890,72899,72900,72901,72902,72903,72904, 72905,72906,72907,72909,73099,73100,73101,73102,73103,73104, 73105,73108,73109,73299,73309,73319,73390,73391,73392,73393, 73399,73400,73401,73402,73408,73409,73419,73490,73491,73499, 73500,73501,73502,73503,73504,73508,73509,73699,73799,73800, 73801,73802,73803,73804,73805,73806,73809
	**ICD10:**	M00,M000,M000A,M000B,M001,M001A,M002,M002A,M002B,M008, M009,M02,M021,M022,M023,M028,M029,M05,M050,M050A, M051,M051B,M051C,M051D,M051E,M051F,M052,M053,M053A,M053C, M053D,M053E,M058,M059,M06,M060,M061,M062,M063,M064, M068,M069,M11,M110,M112,M112A,M118,M119,M12,M120, M121,M122,M123,M124,M128,M128A,M13,M130,M131,M138,

		M138A,M139,M15,M150,M151,M152,M153,M153A,M154,M158,<br>M159,M16,M160,M161,M161A,M161B,M162,M163,M163A,M163B,<br>M166,M167,M167A,M167B,M169,M17,M170,M171,M171A,M171B,<br>M174,M175,M175A,M175B,M179,M18,M180,M181,M181A,M181B,<br>M184,M185,M185A,M185B,M189,M19,M190,M190A,M192,M192A,<br>M198,M198A,M199,M30,M300,M301,M303,M308,M308A,M31,<br>M310,M310A,M310B,M311,M311A,M311B,M312,M313,M314,M315,<br>M315A,M316,M316A,M318,M318A,M318B,M319,M33,M331,M332,<br>M339,M34,M340,M341,M348,M348A,M348B,M348C,M349,M35,<br>M350,M350A,M350B,M350C,M350D,M350E,M351,M351A,M352,M353,<br>M354,M355,M356,M358,M359,M40,M400,M401,M401A,M402,<br>M402A,M403,M404,M404A,M404B,M404C,M405,M41,M412,M413,<br>M413A,M415,M415A,M415B,M418,M419,M42,M421,M429,M43,<br>M430,M431,M432,M432A,M433,M434,M434A,M435,M435A,M436,<br>M438,M439,M45,M459,M459A,M459B,M46,M460,M461,M462,<br>M463,M463A,M464,M465,M465A,M468,M469,M47,M470,M470A,<br>M470B,M471,M471A,M471B,M471C,M472,M472A,M472B,M478,M478A,<br>M478B,M478C,M478D,M478E,M479,M48,M480,M481,M481A,M482,<br>M482A,M483,M484,M485,M485A,M485B,M488,M488A,M489,M50,<br>M500,M500A,M500B,M500C,M500D,M500E,M500F,M501,M501A,M501B,<br>M501C,M501D,M501E,M501F,M502,M503,M508,M509,M51,M510,<br>M510A,M510B,M510C,M510D,M510E,M510F,M510G,M511,M511A,M511B,<br>M511C,M511D,M511E,M511F,M511H,M511I,M512,M512A,M512B,M512C,<br>M512D,M512E,M512F,M512G,M513,M513A,M513B,M513C,M514,M518,<br>M519,M53,M530,M530A,M531,M532,M533,M533A,M533B,M538,<br>M539,M54,M540,M540A,M540B,M541,M541A,M541B,M541C,M541D,<br>M542,M543,M544,M545,M545A,M546,M548,M549,M60,M600,<br>M600A,M601,M602,M602B,M608,M609,M61,M610,M611,M612,<br>M612A,M612B,M613,M614,M615,M619,M619A,M62,M620,M620A,<br>M621,M621A,M622,M623,M624,M625,M625A,M626,M628,M628A,<br>M628B,M629,M65,M650,M651,M652,M653,M653A,M654,M658,<br>M658A,M658B,M659,M659A,M659B,M66,M660,M661,M661A,M662,<br>M663,M664,M665,M67,M670,M671,M672,M673,M674,M678,<br>M679,M71,M710,M711,M712,M713,M713A,M714,M715,M718,<br>M719,M72,M720,M721,M722,M723,M724,M725,M725A,M725B,<br>M728,M728A,M728B,M728C,M729,M75,M750,M751,M751A,M751B,<br>M751C,M752,M753,M753A,M754,M755,M758,M759,M76,M760,<br>M761,M762,M763,M763B,M764,M765,M766,M766A,M766B,M767,<br>M768,M768A,M768B,M769,M77,M770,M771,M772,M773,M774,<br>M775,M778,M779,M779A,M79,M790,M790A,M790B,M791,M792,<br>M792A,M792B,M793,M793A,M793B,M793C,M793F,M794,M795,M796,<br>M797,M798,M798A,M798B,M798C,M798D,M799,M80,M800,M801,<br>M802,M805,M808,M809,M809A,M809B,M809C,M809D,M81,M810,<br>M811,M812,M815,M816,M818,M818A,M819,M83,M830,M831,<br>M832,M832A,M833,M838,M839,M84,M840,M841,M842,M843,<br>M844,M844A,M848,M849,M85,M850,M851,M852,M852A,M853,<br>M854,M855,M856,M858,M858A,M859,M86,M860,M861,M862,<br>M863,M864,M865,M865A,M866,M868,M868A,M869,M869A,M869B,<br>M869C,M87,M870,M873,M878,M879,M88,M880,M888,M889,<br>M89,M890,M890A,M891,M892,M893,M894,M894C,M895,M896,<br>M898,M898A,M898B,M899,M93,M930,M931,M932,M932A,M932B,<br>M938,M939,M94,M940,M941,M942,M943,M948,M948A,M948B,<br>M948D,M948E,M948F,M948G,M949
**neoplasms – all**	**ICD-8:**	14009,14019,14029,14099,14109,14119,14129,14139,14199,14209,

14280,14281,14289,14299,14309,14319,14399,14499,14509,14519,
14589,14599,14609,14689,14699,14700,14701,14702,14708,14709,
14809,14819,14889,14899,14999,15000,15001,15002,15008,15009,
15109,15119,15180,15181,15182,15189,15199,15209,15280,15281,
15289,15299,15300,15301,15302,15309,15319,15329,15339,15380,
15389,15399,15409,15410,15411,15419,15429,15509,15519,15589,
15609,15610,15611,15618,15619,15629,15699,15709,15780,15781,
15789,15799,15809,15899,15999,16009,16019,16029,16089,16099,
16109,16180,16181,16189,16199,16209,16210,16211,16212,16213,
16214,16215,16216,16218,16219,16309,16319,16399,17009,17019,
17029,17039,17049,17059,17069,17079,17089,17099,17109,17119,
17129,17139,17199,17209,17219,17229,17230,17239,17249,17259,
17260,17261,17262,17269,17279,17289,17299,17309,17319,17329,
17330,17339,17349,17359,17360,17361,17362,17369,17379,17389,
17399,17400,17401,17402,17408,17409,18000,18001,18002,18003,
18008,18009,18199,18200,18201,18202,18203,18208,18209,18299,
18300,18301,18302,18303,18308,18309,18319,18399,18400,18401,
18409,18419,18489,18499,18599,18699,18700,18701,18702,18708,
18709,18780,18789,18799,18800,18801,18802,18808,18809,18909,
18919,18929,18990,18991,18992,18999,19000,19001,19002,19003,
19004,19008,19009,19100,19101,19102,19103,19104,19105,19106,
19107,19108,19109,19200,19201,19208,19209,19219,19220,19228,
19229,19239,19249,19259,19299,19399,19409,19419,19429,19439,
19449,19489,19499,19509,19519,19590,19591,19593,19599,19609,
19619,19629,19639,19649,19679,19689,19699,19709,19719,19729,
19739,19749,19759,19769,19779,19789,19799,19809,19810,19819,
19829,19839,19841,19842,19849,19850,19851,19859,19890,19891,
19892,19899,19909,19910,19919,20009,20019,20199,20209,20219,
20220,20229,20290,20299,20399,20409,20419,20499,20509,20519,
20599,20609,20619,20699,20709,20719,20729,20799,20899,20900,
20901,20902,20908,20909,21009,21019,21020,21021,21022,21029,
21039,21040,21041,21042,21049,21059,21069,21079,21089,21099,
21109,21119,21120,21121,21122,21128,21129,21130,21131,21132,
21133,21134,21135,21136,21138,21139,21149,21150,21151,21152,
21158,21159,21169,21170,21171,21179,21199,21200,21201,21202,
21203,21204,21209,21219,21229,21230,21231,21239,21249,21259,
21299,21300,21301,21302,21303,21304,21305,21306,21307,21308,
21309,21499,21500,21501,21502,21503,21508,21509,21600,21601,
21602,21603,21604,21605,21606,21607,21608,21609,21619,21629,
21689,21699,21700,21701,21702,21708,21709,21899,21909,21919,
21990,21991,21999,22099,22100,22101,22102,22109,22119,22129,
22189,22199,22209,22219,22280,22281,22289,22299,22309,22319,
22329,22330,22331,22332,22338,22339,22380,22389,22399,22400,
22401,22402,22403,22404,22408,22409,22500,22501,22502,22503,
22504,22505,22506,22507,22508,22509,22510,22511,22518,22519,
22520,22521,22522,22523,22524,22525,22528,22529,22530,22531,
22539,22549,22559,22569,22599,22609,22619,22620,22621,22629,
22639,22689,22699,22700,22701,22702,22703,22709,22899,23009,
23019,23020,23021,23022,23028,23029,23030,23031,23032,23033,
23034,23035,23036,23038,23039,23049,23050,23051,23052,23058,
23059,23069,23079,23099,23101,23102,23103,23104,23108,23109,
23119,23129,23130,23131,23139,23149,23159,23199,23200,23201,
23202,23203,23204,23205,23206,23208,23209,23210,23211,23212,
23213,23218,23219,23220,23221,23222,23223,23224,23225,23226,

		23227,23228,23229,23300,23301,23302,23308,23309,23409,23419, 23490,23499,23590,23599,23609,23610,23618,23619,23620,23628, 23629,23689,23699,23709,23719,23720,23721,23729,23739,23749, 23759,23769,23799,23800,23801,23802,23803,23804,23808,23809, 23819,23829,23839,23849,23859,23869,23879,23899,23909,23919, 23999
	**ICD10:**	C00,C000,C001,C002,C003,C004,C005,C006,C008, C009,C01,C019,C02,C020,C021,C021A,C021B,C022,C022A, C023,C024,C028,C029,C03,C030,C031,C039,C04,C040, C041,C048,C049,C05,C050,C051,C052,C058,C059,C06, C060,C061,C062,C068,C069,C07,C079,C08,C080,C081, C088,C089,C09,C090,C090A,C090B,C091,C091A,C091B,C092, C098,C099,C10,C100,C101,C102,C103,C104,C104A,C108, C109,C11,C110,C111,C112,C113,C118,C119,C12,C129, C13,C130,C131,C132,C138,C139,C14,C140,C141,C142, C148,C15,C150,C151,C152,C153,C154,C155,C158,C159, C16,C160,C161,C162,C163,C164,C165,C166,C168,C169, C17,C170,C171,C172,C173,C178,C179,C18,C180,C180A, C181,C182,C183,C184,C185,C186,C187,C188,C188A,C189, C19,C199,C20,C209,C21,C210,C210D,C210H,C210J,C210K, C210L,C210M,C210R,C210V,C210W,C210Z,C211,C212,C218,C22, C220,C221,C222,C223,C224,C227,C229,C23,C239,C24, C240,C240A,C240B,C240C,C240D,C240E,C241,C248,C249,C25, C250,C251,C252,C253,C254,C257,C258,C259,C26,C260, C261,C268,C269,C30,C300,C300A,C300B,C300C,C301,C301B, C31,C310,C311,C312,C313,C318,C319,C32,C320,C320A, C321,C321A,C321B,C321C,C321D,C322,C323,C328,C329,C33, C339,C34,C340,C340A,C341,C342,C343,C348,C349,C37, C379,C38,C380,C380A,C381,C382,C383,C384,C388,C39, C390,C398,C399,C40,C400,C400C,C400D,C401,C402,C402B, C403,C403A,C403B,C408,C408B,C409,C409B,C41,C410,C410A, C410B,C410D,C411,C412,C412A,C413,C413A,C413E,C413F,C414, C414E,C414F,C418,C418B,C419,C419B,C43,C430,C430E,C430F, C431,C431A,C431B,C431F,C431J,C432,C432A,C432F,C432Z,C433, C433E,C433F,C433G,C433J,C434,C434A,C434B,C434E,C434F,C434G, C434K,C434Z,C435,C435A,C435B,C435C,C435D,C435E,C435F,C435G, C435Z,C436,C436B,C436C,C436E,C436F,C436G,C436Z,C437,C437A, C437B,C437C,C437E,C437F,C437G,C437H,C437J,C437Z,C438,C438E, C438F,C438G,C438Z,C439,C439F,C439G,C439J,C439K,C439Z,C44, C440,C440D,C440E,C440F,C440H,C440J,C440K,C440Q,C440T,C440V, C440Z,C441,C441A,C441B,C441D,C441E,C441F,C441G,C441H,C441J, C441K,C441T,C441Z,C442,C442A,C442B,C442C,C442D,C442E,C442F, C442H,C442J,C442K,C442L,C442M,C442P,C443,C443A,C443D,C443E, C443F,C443G,C443H,C443J,C443K,C443M,C443P,C443R,C443S,C443Z, C444,C444A,C444B,C444D,C444E,C444F,C444G,C444H,C444J,C444K, C444M,C444P,C444S,C444V,C444W,C444Z,C445,C445A,C445B,C445C, C445D,C445E,C445F,C445G,C445H,C445J,C445K,C445L,C445M,C445N, C445P,C445R,C445U,C445V,C445W,C445Z,C446,C446A,C446B,C446C, C446D,C446E,C446G,C446H,C446J,C446K,C446L,C446M,C446P,C446Z, C447,C447B,C447C,C447D,C447E,C447F,C447G,C447H,C447J,C447K, C447L,C447M,C447P,C447R,C447V,C447W,C447Z,C448,C448D,C448E, C448F,C448G,C448H,C448K,C448P,C448W,C449,C449D,C449E,C449F, C449G,C449H,C449J,C449K,C449L,C449M,C449N,C449P,C449Q,C449R, C449T,C449V,C449Z,C45,C450,C451,C452,C457,C459,C46,

C460,C461,C462,C463,C467,C468,C469,C47,C470,C471,
C471A,C472,C473,C473A,C474,C474B,C475,C476,C478,C479,
C48,C480,C481,C481A,C481B,C481C,C482,C488,C49,C490,
C490A,C490B,C490C,C491,C491A,C491B,C492,C492A,C492B,C493,
C493A,C494,C495,C495A,C496,C498,C498A,C499,C50,C500,
C500A,C500B,C500C,C500D,C501,C502,C503,C504,C505,C506,
C508,C509,C51,C510,C510A,C511,C512,C518,C519,C519E,
C519H,C519K,C519M,C519R,C519Z,C52,C529,C53,C530,C531,
C532,C533,C534,C538,C539,C54,C540,C541,C542,C543,
C544,C545,C546,C548,C549,C55,C559,C56,C560,C561,
C562,C563,C569,C57,C570,C571,C572,C573,C574,C577,
C578,C579,C58,C589,C60,C600,C601,C602,C608,C609,
C609D,C609J,C609R,C609Z,C61,C619,C62,C620,C621,C629,
C629A,C63,C630,C631,C632,C637,C638,C639,C64,C649,
C65,C659,C66,C669,C67,C670,C671,C672,C673,C674,
C675,C675A,C676,C677,C678,C679,C68,C680,C681,C688,
C689,C69,C690,C691,C692,C692A,C693,C694,C694A,C695,
C695A,C695B,C696,C697,C698,C699,C70,C700,C701,C709,
C71,C710,C710A,C710B,C711,C712,C713,C714,C715,C716,
C717,C717A,C717B,C717C,C718,C719,C72,C720,C721,C722,
C722A,C723,C724,C725,C728,C729,C73,C739,C74,C740,
C741,C749,C75,C750,C751,C752,C753,C754,C755,C755B,
C758,C759,C76,C760,C760A,C760B,C760C,C761,C762,C763,
C764,C765,C767,C768,C77,C770,C770A,C770B,C770C,C770D,
C770E,C770F,C770G,C770H,C771,C771A,C771B,C772,C772A,C772B,
C773,C773A,C773B,C773C,C773D,C774,C774A,C774B,C774C,C774D,
C775,C775A,C775B,C778,C778A,C778B,C779,C779A,C779B,C78,
C780,C781,C782,C783,C784,C785,C785A,C785B,C786,C786A,
C786B,C786C,C787,C788,C79,C790,C790A,C790B,C791,C791I,
C791J,C791S,C791T,C791U,C791V,C791X,C792,C793,C793A,C793B,
C793C,C794,C795,C795A,C795B,C796,C797,C798,C80,C809,
C81,C810,C811,C812,C813,C817,C819,C82,C820,C821,
C822,C827,C829,C83,C830,C831,C832,C833,C834,C835,
C836,C837,C838,C839,C84,C840,C840A,C840B,C840C,C841,
C842,C843,C844,C844A,C844D,C844E,C845,C845A,C845B,C845D,
C85,C850,C851,C851A,C851B,C851C,C851D,C851E,C857,C857A,
C859,C859A,C859B,C88,C880,C881,C882,C883,C887,C889,
C90,C900,C901,C902,C902A,C91,C910,C911,C912,C913,
C914,C914A,C915,C917,C917A,C919,C92,C920,C921,C922,
C923,C923B,C924,C925,C927,C927A,C929,C93,C930,C931,
C932,C937,C939,C94,C940,C941,C942,C943,C943A,C944,
C945,C947,C95,C950,C950A,C950B,C951,C952,C957,C959,
C96,C960,C961,C962,C963,C967,C967A,C969,C97,C979,
D00,D000,D000A,D000B,D000C,D001,D002,D01,D010,D011,
D012,D013,D013A,D013B,D014,D015,D015A,D015B,D017,D019,
D02,D020,D021,D022,D022B,D023,D023B,D024,D03,D030,
D031,D031B,D031E,D032,D032E,D033,D033E,D033F,D034,D034A,
D034B,D034E,D034F,D035,D035A,D035B,D035C,D035E,D035F,D036,
D036A,D036B,D036E,D036F,D037,D037A,D037E,D038,D038E,D038F,
D039,D039E,D039F,D04,D040,D040E,D040Z,D041,D041A,D041B,
D041D,D041Z,D042,D042A,D042B,D042D,D043,D043A,D043B,D043D,
D043Z,D044,D044A,D044B,D044D,D044Z,D045,D045A,D045B,D045C,
D045D,D045E,D045Z,D046,D046B,D046D,D046G,D046Z,D047,D047A,
D047D,D047Z,D048,D048D,D048E,D048Z,D049,D049D,D049E,D049Z,

D05,D050,D051,D057,D059,D06,D060,D061,D067,D069,
D07,D070,D071,D071D,D071E,D071G,D071Z,D072,D073,D074,
D074D,D074E,D074Z,D075,D076,D076A,D076T,D09,D090,D091,
D092,D093,D093A,D097,D099,D10,D100,D101,D101A,D102,
D103,D103A,D103B,D104,D104A,D105,D106,D107,D109,D11,
D110,D117,D117A,D117B,D119,D12,D120,D120A,D121,D122,
D123,D123A,D123B,D124,D125,D126,D126A,D126B,D126C,D126F,
D127,D128,D129,D129A,D129B,D13,D130,D131,D132,D133,
D133A,D134,D134A,D135,D136,D137,D137A,D137B,D139,D14,
D140,D140A,D140B,D140C,D141,D142,D143,D143A,D143B,D144,
D15,D150,D151,D152,D157,D159,D16,D160,D160A,D160C,
D160D,D161,D161A,D161B,D162,D162A,D162B,D163,D163A,D163B,
D163C,D164,D164A,D164C,D164D,D165,D165A,D166,D166B,D167,
D167A,D167B,D167D,D167E,D167F,D168,D168B,D168D,D168E,D168F,
D169,D169A,D169B,D17,D170,D170A,D170B,D170C,D170D,D170E,
D170F,D171,D171A,D171B,D172,D172A,D172B,D173,D173A,D173B,
D174,D175,D176,D177,D177A,D177B,D179,D18,D180,D180A,
D180B,D180C,D180D,D180E,D180F,D181,D181A,D181B,D189,D19,
D190,D191,D192,D192D,D193,D194,D195,D196,D197,D199,
D20,D200,D201,D21,D210,D210A,D210B,D210C,D211,D211A,
D211B,D212,D212A,D212B,D213,D214,D215,D216,D217,D217A,
D219,D22,D220,D220D,D220E,D220G,D220P,D220Z,D221,D221A,
D221B,D221D,D221G,D221N,D221P,D222,D222A,D222B,D222D,D222F,
D222G,D222P,D223,D223A,D223D,D223E,D223F,D223G,D223H,D223K,
D223L,D223N,D223P,D223Q,D223V,D223Z,D224,D224A,D224B,D224D,
D224E,D224F,D224G,D224L,D224P,D224Q,D224V,D225,D225B,D225D,
D225E,D225F,D225G,D225H,D225K,D225L,D225M,D225P,D225Q,D225V,
D225Z,D226,D226B,D226D,D226E,D226F,D226G,D226N,D226P,D226V,
D226Z,D227,D227A,D227B,D227D,D227E,D227F,D227G,D227H,D227P,
D227V,D228,D228A,D228D,D228E,D228G,D228H,D228L,D228N,D228P,
D228Q,D229,D229D,D229E,D229F,D229G,D229H,D229K,D229L,D229M,
D229N,D229P,D229Q,D229V,D229Z,D23,D230,D2301,D2308,D230D,
D230F,D230H,D230J,D230K,D230L,D230Q,D230T,D230U,D230X,D231,
D2318,D231A,D231B,D231D,D231F,D231H,D231I,D231J,D231K,D231L,
D231Q,D231T,D231X,D232,D2328,D232A,D232B,D232D,D232F,D232H,
D232J,D232L,D232N,D232Q,D232R,D232X,D233,D2331,D2338,D233D,
D233F,D233G,D233H,D233I,D233L,D233Q,D233R,D233T,D233X,D233Y,
D233Z,D234,D2341,D2348,D234A,D234B,D234D,D234F,D234G,D234H,
D234I,D234J,D234L,D234N,D234Q,D234R,D234T,D234X,D235,D2351,
D2358,D235A,D235B,D235C,D235D,D235H,D235I,D235J,D235K,D235L,
D235M,D235N,D235Q,D235R,D235T,D235X,D235Y,D236,D2361,D2368,
D236B,D236D,D236E,D236H,D236I,D236J,D236K,D236L,D236N,D236R,
D236X,D237,D2371,D2378,D237A,D237B,D237D,D237E,D237H,D237J,
D237K,D237L,D237M,D237N,D237R,D237S,D237X,D237Y,D238,D2381,
D2388,D238D,D238E,D238G,D238H,D238J,D238L,D238N,D238Q,D238R,
D238X,D238Y,D238Z,D239,D2391,D2392,D2398,D239A,D239D,D239E,
D239G,D239H,D239I,D239J,D239K,D239L,D239M,D239N,D239Q,D239R,
D239T,D239X,D239Z,D24,D249,D249A,D249B,D249C,D249D,D249E,
D249F,D249G,D249W,D25,D250,D250A,D250B,D250C,D251,D252,
D259,D26,D260,D261,D267,D269,D27,D270,D271,D272,
D278,D279,D28,D280,D281,D282,D282A,D282C,D287,D289,
D2891,D2898,D289H,D289M,D289N,D289Q,D289R,D289X,D29,D290,
D291,D292,D293,D294,D297,D297A,D297C,D299,D2998,D299J,
D299K,D299L,D299M,D299Q,D299T,D299X,D30,D300,D301,D302,

		D303,D304,D307,D309,D31,D310,D311,D312,D313,D314,D315,D315A,D315B,D316,D316A,D317,D319,D32,D320,D321,D329,D329A,D33,D330,D330A,D330B,D330C,D330D,D331,D331A,D331B,D331C,D331D,D332,D333,D333A,D333B,D334,D337,D339,D34,D349,D35,D350,D350A,D351,D352,D352A,D353,D354,D355,D356,D356B,D356C,D357,D358,D359,D36,D360,D361,D361A,D367,D369,D37,D370,D370A,D370B,D370C,D371,D372,D373,D374,D375,D375A,D376,D376A,D376B,D376C,D377,D379,D38,D380,D381,D381A,D381B,D381C,D382,D383,D384,D385,D385A,D385B,D385C,D389,D39,D390,D391,D391A,D392,D392A,D392B,D397,D399,D40,D400,D401,D407,D409,D41,D410,D411,D412,D413,D414,D417,D419,D42,D420,D421,D429,D43,D430,D430B,D430C,D430D,D430E,D430F,D431,D431A,D431B,D431C,D431D,D431E,D431F,D432,D433,D434,D437,D439,D44,D440,D441,D442,D443,D444,D445,D446,D447,D447B,D448,D449,D45,D459,D46,D460,D461,D462,D463,D464,D467,D469,D47,D470,D470A,D471,D471A,D472,D473,D474,D477,D477A,D479,D48,D480,D481,D482,D483,D484,D485,D486,D487,D487A,D487B,D487C,D487D,D489
**neoplasms – benign**	**ICD-8:**	21009,21019,21020,21021,21022,21029,21039,21040,21041,21042,21049,21059,21069,21079,21089,21099,21109,21119,21120,21121,21122,21128,21129,21130,21131,21132,21133,21134,21135,21136,21138,21139,21149,21150,21151,21152,21158,21159,21169,21170,21171,21179,21199,21200,21201,21202,21203,21204,21209,21219,21229,21230,21231,21239,21249,21259,21299,21300,21301,21302,21303,21304,21305,21306,21307,21308,21309,21499,21500,21501,21502,21503,21508,21509,21600,21601,21602,21603,21604,21605,21606,21607,21608,21609,21619,21629,21689,21699,21700,21701,21702,21708,21709,21899,21909,21919,21990,21991,21999,22099,22100,22101,22102,22109,22119,22129,22189,22199,22209,22219,22280,22281,22289,22299,22309,22319,22329,22330,22331,22332,22338,22339,22380,22389,22399,22400,22401,22402,22403,22404,22408,22409,22500,22501,22502,22503,22504,22505,22506,22507,22508,22509,22510,22511,22518,22519,22520,22521,22522,22523,22524,22525,22528,22529,22530,22531,22539,22549,22559,22569,22599,22609,22619,22620,22621,22629,22639,22689,22699,22700,22701,22702,22703,22709,22899
	**ICD10:**	D10,D100,D101,D101A,D102,D103,D103A,D103B,D104,D104A,D105,D106,D107,D109,D11,D110,D117,D117A,D117B,D119,D12,D120,D120A,D121,D122,D123,D123A,D123B,D124,D125,D126,D126A,D126B,D126C,D126F,D127,D128,D129,D129A,D129B,D13,D130,D131,D132,D133,D133A,D134,D134A,D135,D136,D137,D137A,D137B,D139,D14,D140,D140A,D140B,D140C,D141,D142,D143,D143A,D143B,D144,D15,D150,D151,D152,D157,D159,D16,D160,D160A,D160C,D160D,D161,D161A,D161B,D162,D162A,D162B,D163,D163A,D163B,D163C,D164,D164A,D164C,D164D,D165,D165A,D166,D166B,D167,D167A,D167B,D167D,D167E,D167F,D168,D168B,D168D,D168E,D168F,D169,D169A,D169B,D17,D170,D170A,D170B,D170C,D170D,D170E,D170F,D171,D171A,D171B,D172,D172A,D172B,D173,D173A,D173B,D174,D175,D176,D177,D177A,D177B,D179,D18,D180,D180A,D180B,D180C,D180D,D180E,D180F,D181,D181A,D181B,D189,D19,D190,D191,D192,D192D,D193,D194,D195,D196,D197,D199,D20,D200,D201,D21,D210,D210A,D210B,D210C,D211,D211A,

		D211B,D212,D212A,D212B,D213,D214,D215,D216,D217,D217A,<br>D219,D22,D220,D220D,D220E,D220G,D220P,D220Z,D221,D221A,<br>D221B,D221D,D221G,D221N,D221P,D222,D222A,D222B,D222D,D222F,<br>D222G,D222P,D223,D223A,D223D,D223E,D223F,D223G,D223H,D223K,<br>D223L,D223N,D223P,D223Q,D223V,D223Z,D224,D224A,D224B,D224D,<br>D224E,D224F,D224G,D224L,D224P,D224Q,D224V,D225,D225B,D225D,<br>D225E,D225F,D225G,D225H,D225K,D225L,D225M,D225P,D225Q,D225V,<br>D225Z,D226,D226B,D226D,D226E,D226F,D226G,D226N,D226P,D226V,<br>D226Z,D227,D227A,D227B,D227D,D227E,D227F,D227G,D227H,D227P,<br>D227V,D228,D228A,D228D,D228E,D228G,D228H,D228L,D228N,D228P,<br>D228Q,D229,D229D,D229E,D229F,D229G,D229H,D229K,D229L,D229M,<br>D229N,D229P,D229Q,D229V,D229Z,D23,D230,D2301,D2308,D230D,<br>D230F,D230H,D230J,D230K,D230L,D230Q,D230T,D230U,D230X,D231,<br>D2318,D231A,D231B,D231D,D231F,D231H,D231I,D231J,D231K,D231L,<br>D231Q,D231T,D231X,D232,D2328,D232A,D232B,D232D,D232F,D232H,<br>D232J,D232L,D232N,D232Q,D232R,D232X,D233,D2331,D2338,D233D,<br>D233F,D233G,D233H,D233I,D233L,D233Q,D233R,D233T,D233X,D233Y,<br>D233Z,D234,D2341,D2348,D234A,D234B,D234D,D234F,D234G,D234H,<br>D234I,D234J,D234L,D234N,D234Q,D234R,D234T,D234X,D235,D2351,<br>D2358,D235A,D235B,D235C,D235D,D235H,D235I,D235J,D235K,D235L,<br>D235M,D235N,D235Q,D235R,D235T,D235X,D235Y,D236,D2361,D2368,<br>D236B,D236D,D236E,D236H,D236I,D236J,D236K,D236L,D236N,D236R,<br>D236X,D237,D2371,D2378,D237A,D237B,D237D,D237E,D237H,D237J,<br>D237K,D237L,D237M,D237N,D237R,D237S,D237X,D237Y,D238,D2381,<br>D2388,D238D,D238E,D238G,D238H,D238J,D238L,D238N,D238Q,D238R,<br>D238X,D238Y,D238Z,D239,D2391,D2392,D2398,D239A,D239D,D239E,<br>D239G,D239H,D239I,D239J,D239K,D239L,D239M,D239N,D239Q,D239R,<br>D239T,D239X,D239Z,D24,D249,D249A,D249B,D249C,D249D,D249E,<br>D249F,D249G,D249W,D25,D250,D250A,D250B,D250C,D251,D252,<br>D259,D26,D260,D261,D267,D269,D27,D270,D271,D272,<br>D278,D279,D28,D280,D281,D282,D282A,D282C,D287,D289,<br>D2891,D2898,D289H,D289M,D289N,D289Q,D289R,D289X,D29,D290,<br>D291,D292,D293,D294,D297,D297A,D297C,D299,D2998,D299J,<br>D299K,D299L,D299M,D299Q,D299T,D299X,D30,D300,D301,D302,<br>D303,D304,D307,D309,D31,D310,D311,D312,D313,D314,<br>D315,D315A,D315B,D316,D316A,D317,D319,D32,D320,D321,<br>D329,D329A,D33,D330,D330A,D330B,D330C,D330D,D331,D331A,<br>D331B,D331C,D331D,D332,D333,D333A,D333B,D334,D337,D339,<br>D34,D349,D35,D350,D350A,D351,D352,D352A,D353,D354,<br>D355,D356,D356B,D356C,D357,D358,D359,D36,D360,D361,<br>D361A,D367,D369
**circulatory – all**	**ICD-8:**	39099,39109,39119,<br>39129,39199,39209,39299,39300,39301,39308,39309,39400,39401,<br>39402,39408,39409,39490,39491,39492,39498,39499,39500,39501,<br>39502,39508,39509,39590,39591,39592,39598,39599,39600,39601,<br>39602,39603,39604,39608,39609,39690,39691,39692,39693,39694,<br>39698,39699,39700,39701,39709,39899,40009,40019,40029,40039,<br>40099,40199,40299,40399,40499,41009,41099,41109,41199,41209,<br>41299,41309,41399,41409,41499,42000,42001,42008,42009,42100,<br>42101,42108,42109,42199,42299,42300,42301,42302,42308,42309,<br>42400,42401,42402,42408,42409,42410,42411,42412,42418,42419,<br>42490,42491,42492,42499,42599,42600,42601,42602,42608,42609,<br>42709,42710,42711,42719,42720,42721,42722,42723,42724,42725,<br>42726,42727,42728,42729,42790,42791,42792,42793,42794,42795,

		42796,42797,42799,42899,42900,42908,42909,43000,43001,43008,43009,43090,43091,43098,43099,43100,43101,43108,43109,43190,43191,43198,43199,43200,43201,43202,43208,43209,43290,43291,43292,43298,43299,43309,43399,43409,43499,43509,43599,43600,43601,43609,43690,43699,43700,43701,43708,43709,43790,43791,43798,43799,43809,43899,44009,44019,44020,44021,44028,44029,44030,44039,44099,44109,44110,44111,44119,44120,44121,44129,44199,44299,44300,44301,44302,44308,44309,44319,44329,44380,44381,44382,44389,44399,44400,44408,44409,44419,44420,44421,44428,44429,44439,44440,44441,44442,44443,44444,44448,44449,44490,44499,44609,44619,44629,44630,44631,44639,44649,44690,44691,44692,44699,44799,44801,44802,44808,44809,044990,
	**ICD10:**	I00,I009,I009A,I009B,I01,I010,I011,I012,I018,I018A,I019,I02,I020,I020A,I029,I05,I050,I051,I052,I058,I059,I06,I060,I061,I062,I068,I069,I07,I070,I071,I072,I078,I079,I08,I080,I080A,I081,I081A,I082,I082A,I083,I088,I089,I09,I090,I091,I091A,I091B,I092,I092B,I092C,I098,I098A,I098B,I098E,I099,I10,I109,I11,I110,I119,I12,I120,I129,I13,I130,I131,I132,I139,I15,I150,I151,I152,I158,I159,I20,I200,I200A,I200B,I200C,I201,I201A,I201B,I208,I208A,I208B,I208D,I209,I21,I210,I210A,I210B,I211,I211A,I211B,I212,I212A,I212B,I212C,I212E,I212G,I212H,I213,I214,I219,I22,I220,I220A,I220C,I221,I221A,I221B,I228,I228B,I228C,I228F,I228G,I229,I23,I230,I231,I232,I233,I234,I235,I236,I236A,I236B,I238,I238A,I24,I240,I240A,I241,I241A,I248,I248A,I249,I25,I250,I251,I251A,I251B,I251C,I252,I252A,I252B,I252C,I253,I254,I255,I256,I256A,I258,I259,I26,I260,I260A,I269,I269A,I27,I270,I271,I272,I278,I279,I279A,I28,I280,I281,I288,I288A,I288B,I289,I30,I300,I301,I301A,I301B,I301C,I301E,I308,I309,I31,I310,I310B,I311,I311A,I311B,I312,I313,I313A,I318,I318B,I319,I319A,I33,I330,I330A,I330B,I330C,I330D,I330E,I330F,I339,I34,I340,I341,I342,I348,I348A,I349,I35,I350,I351,I352,I358,I358A,I359,I36,I360,I361,I362,I368,I368A,I369,I37,I370,I371,I372,I378,I379,I38,I389,I40,I400,I400A,I400B,I401,I408,I409,I42,I420,I421,I421A,I422,I423,I423B,I424,I424A,I425,I428,I428A,I428B,I429,I44,I440,I441,I441A,I441B,I441C,I441D,I441E,I442,I442A,I443,I443A,I444,I445,I446,I446A,I446B,I447,I45,I450,I451,I451A,I452,I453,I454,I455,I455A,I455B,I455C,I455G,I455H,I455K,I455L,I456,I456A,I456AA,I456B,I456C,I456D,I456DA,I456DB,I456E,I458,I458M,I459,I459A,I46,I460,I461,I469,I47,I470,I470A,I470B,I470C,I470D,I470H,I470HA,I470HB,I471,I471A,I471B,I471C,I471E,I471EA,I471EB,I471F,I471FA,I471G,I471H,I471J,I471L,I471LA,I471M,I471N,I471P,I471PA,I471PB,I471R,I471RA,I471RB,I471RC,I471X,I472,I472A,I472B,I472D,I472E,I472EA,I472F,I472FA,I472H,I472L,I472LA,I472LB,I472LC,I472M,I472N,I472NA,I478,I478A,I479,I479A,I48,I489,I489A,I489AA,I489AB,I489AC,I489AD,I489AE,I489B,I489BA,I489BB,I489BC,I489BD,I49,I490,I490A,I490B,I491,I491A,I491B,I491C,I491D,I491E,I492,I493,I493A,I493B,I493C,I494,I494A,I495,I495A,I495B,I498,I498A,I498B,I498C,I498D,I498E,I499,I499A,I50,I500,I500A,I501,I501A,I501B,I501C,I502,I503,I508,I508A,I509,I509A,I509B,I51,I510,I510A,I510B,I511,I511A,I512,

		I513,I513A,I513B,I513C,I514,I514A,I515,I516,I517,I517A,I517B,I517C,I518,I518A,I519,I60,I600,I601,I602,I603,I604,I605,I606,I606A,I606B,I606C,I606D,I607,I607A,I608,I609,I61,I610,I610A,I611,I611A,I611B,I612,I613,I614,I615,I616,I618,I619,I62,I620,I621,I629,I63,I630,I631,I632,I633,I634,I634A,I635,I636,I638,I639,I64,I649,I65,I650,I650A,I650B,I651,I651A,I651B,I652,I652A,I652B,I653,I653B,I653C,I653D,I658,I659,I66,I660,I660A,I660B,I661,I662,I662A,I663,I664,I668,I668A,I669,I67,I670,I671,I671A,I672,I672A,I673,I673A,I674,I676,I676A,I677,I678,I678A,I678B,I679,I69,I690,I691,I692,I693,I694,I698,I70,I700,I701,I702,I702A,I702B,I708,I709,I71,I710,I710A,I710B,I711,I712,I713,I714,I715,I716,I718,I719,I719A,I719B,I72,I720,I721,I722,I723,I724,I728,I729,I73,I730,I731,I738,I738A,I738B,I738C,I738D,I739,I739A,I739B,I739C,I74,I740,I740A,I740B,I740C,I740D,I741,I741A,I741B,I742,I742A,I742B,I743,I743A,I743B,I744,I744A,I744B,I744C,I744D,I744E,I745,I745A,I745B,I748,I749,I77,I770,I770A,I771,I772,I772A,I772B,I773,I774,I775,I776,I776A,I778,I778A,I779,I78,I781,I781A,I781B,I781C,I781D,I788,I788A,I788B,I788C,I788D,I789
**nervous – all**	**ICD-8:**	32009,32019,32080,32089,32090,32091,32092,32093,32099,32199,32200,32201,32202,32203,32204,32205,32208,32209,32300,32301,32302,32303,32308,32309,32400,32401,32408,32409,34000,34001,34008,34009,34100,34101,34109,34299,34600,34601,34608,34609,34790,34791,34792,34793,34794,34795,34796,34799,35799,35800,35801,35808,35809
	**ICD10:**	G00,G000,G001,G002,G003,G008,G008A,G008B,G009,G009A,G03,G030,G031,G032,G038,G039,G04,G040,G040A,G041,G042,G042A,G048,G048A,G048B,G048C,G049,G049A,G049B,G049C,G06,G060,G060B,G060C,G060D,G060E,G060F,G060G,G060I,G060J,G060M,G061,G061A,G061B,G061C,G061E,G062,G062A,G062B,G062C,G08,G089,G089A,G089B,G089L,G09,G099,G20,G209,G209A,G21,G210,G211,G212,G213,G218,G218A,G219,G23,G231,G231A,G232,G238,G239,G30,G300,G301,G308,G309,G31,G310,G310A,G310B,G311,G318,G318B,G318D,G318E,G319,G35,G359,G359A,G359B,G359C,G36,G360,G360A,G361,G368,G369,G37,G370,G370A,G371,G372,G373,G373A,G374,G375,G378,G379,G54,G540,G540A,G540B,G540C,G541,G542,G543,G544,G545,G545A,G545B,G546,G547,G547A,G548,G549,G56,G560,G561,G562,G562A,G563,G564,G568,G568A,G569,G57,G570,G571,G571A,G572,G573,G574,G575,G576,G576A,G578,G578A,G579,G58,G580,G587,G588,G589,G59,G590,G598,G61,G610,G610A,G611,G618,G619,G64,G649,G649A,G90,G900,G900A,G902,G903,G903A,G903B,G908,G909,G93,G930,G930A,G930B,G931,G932,G933,G933A,G934,G935,G935A,G935B,G935C,G936,G937,G939,G95,G950,G950A,G950B,G951,G951A,G951B,G951D,G951G,G952,G952A,G958,G958A,G958B,G958C,G959,G96,G960,G960A,G960B,G961,G968,G969,G98,G989
**mental – all**	**ICD-8:**	29009,29010,29011,29018,29019,29209,29219,29229,29239,29299,29309,29319,29329,29339,29349,29399,29409,29419,29429,29489,29499,29509,

		29519,29529,29539,29559,29569,29589,29599,29709,29719,29799, 29839,29689,30183,29609,29619,29629,29639,29699,29809,29819, 30019,30049,30119,29829,30009,30029,30039,30059,30069,30079, 30089,30099,30509,30519,30529,30539,30549,30559,30560,30568, 30569,30109,30129,30139,30149,30159,30169,30179,30180,30181, 30182,30184,30189,30199
	**ICD10:**	F00,F000,F0000,F0001,F0002,F0003,F0004,F001,F0010,F0011, F0012,F0013,F0014,F002,F0020,F0021,F0022,F0023,F0024,F009, F0090,F0091,F0092,F0093,F0094,F01,F010,F0100,F0101,F0102, F0103,F0104,F011,F0110,F0111,F0112,F0113,F0114,F012,F0120, F0121,F0122,F0123,F0124,F013,F0130,F0131,F0132,F0133,F0134, F018,F0180,F0181,F0182,F0183,F0184,F019,F0190,F0191,F0192, F0193,F0194,F03,F039,F0390,F0391,F0392,F0393,F0394,F04, F049,F05,F050,F051,F058,F059,F09,F099,F20,F200, F2000,F2001,F2002,F2003,F2004,F2005,F2006,F2007,F2008,F2009, F201,F2010,F2011,F2012,F2013,F2014,F2015,F2018,F2019,F202, F2020,F2021,F2022,F2023,F2024,F2025,F2028,F2029,F203,F2030, F2031,F2032,F2033,F2034,F2035,F2038,F2039,F204,F2040,F2044, F205,F2050,F2051,F2052,F2053,F2054,F2055,F2058,F2059,F206, F2060,F2061,F2062,F2063,F2064,F2065,F2069,F208,F2080,F2081, F2084,F2088,F209,F2090,F2091,F2092,F2093,F2094,F2095,F2098, F2099,F21,F210,F2100,F219,F22,F220,F2200,F222,F228, F229,F25,F250,F2500,F2501,F251,F2510,F2511,F252,F2520, F2521,F258,F2580,F2581,F259,F2590,F2591,F28,F280,F289, F29,F299,F30,F300,F301,F302,F3020,F3021,F308,F309, F31,F310,F311,F312,F3120,F3121,F313,F3130,F3131,F314, F315,F3150,F3151,F316,F317,F318,F319,F32,F320,F3200, F3201,F321,F3210,F3211,F322,F323,F3230,F3231,F328,F329, F329A,F33,F330,F3300,F3301,F331,F3310,F3311,F332,F3321, F333,F3330,F3331,F334,F338,F339,F34,F340,F3400,F3401, F341,F3410,F3411,F348,F349,F38,F380,F3800,F381,F3810, F388,F39,F399,F40,F400,F4000,F4001,F401,F402,F402A, F408,F409,F41,F410,F4100,F4101,F411,F412,F413,F418, F419,F42,F420,F421,F422,F4220,F4222,F428,F429,F43, F430,F4300,F4301,F4302,F431,F432,F4320,F4321,F4322,F4323, F4324,F4325,F4328,F433,F4330,F438,F439,F44,F440,F441, F442,F443,F444,F445,F446,F447,F448,F4480,F4481,F4482, F4488,F449,F45,F450,F451,F452,F452A,F453,F4530,F4531, F4532,F4533,F4534,F4538,F454,F458,F459,F48,F480,F481, F488,F489,F60,F600,F601,F602,F6020,F603,F6030,F6031, F604,F605,F606,F607,F608,F609,F6090,F61,F610,F611, F619,F68,F680,F681,F688,F69,F690,F699

B. COVARIATE EFFECTS [PRE-EXISTING CONDITIONS]		
**pre-existing maternal hypertension** – essential (primary) hypertension, hypertensive heart disease, hypertensive renal disease, hypertensive heart and renal disease, secondary hypertension	**ICD-8:**	40009, 40019, 40029, 40039, 40099, 40199, 40299, 40399, 40499
	**ICD10:**	O10, O100, O100A-0C, O101, O101A-1C, O102, O102A-2C, O103, O103A, O104, O104A-4C, O109, O109A-9C, O11, O119, O119A-9C, I10, I109, I11, I110, I119, I12, I120, I129, I13, I130-2, I139, I15, I150-2, I158-9
**pre-existing maternal diabetes** – type I (insulin dependent) and type II (non-insulin dependent), malnutrition-related, other, unspecified	**ICD-8:**	24900-09, 25000-09
	**ICD10:**	E10, E100, E100A-0F, E101-5, E105A-5D, E106-9, E109A, E11, E110, E110A-0E, E111-5, E115A-5D, E116-9, E119A, E12, E120, E120A-0E, E121-5, E125A-5C, E126-9, E13, E130-9, E14, E140, E140A-0D, E141-5, E145A-5D, E146-9, O240, O240A-0C, O241, O241A-1C, O242, O242A, O243, O243A-3B
**B. COVARIATE EFFECTS [PREGNANCY-INDUCED]**		
**maternal bleeding** – haemorrhage in early pregnancy, placenta praevia, premature separation of placenta, antepartum haemorrhage	**ICD-8:**	63209, 63219, 63229, 63239, 63249, 63299, 65100-22, 65124-43, 65145-60, 65163, 65165-66, 65168-80, 65182, 65185-87, 65189-99
	**ICD10:**	O20, O200, O208, O208A, O208B, O209, O44, O440-3, O449, O45, O450, O450D, O451, O451B, O452-3, O458-9, O46, O460, O468, O468A, O469
**fetal oxygen deprivation** – intrauterine hypoxia, birth asphyxia	**ICD-8:**	77639, 77649, 77690-93, 77698-99
	**ICD10:**	P20, P200, P200A-0E, P201, P201A-1B, P201D-1E, P209, P21, P210, P210A-0D, P211, P211A-1D, P219
**pregnancy oedema**	**ICD-8:**	63702
	**ICD10:**	O12, O120-2
