# Supplementary material for: Childhood appendectomy is linked with higher digestive, respiratory, and genitourinary disease risk but lower inflammatory bowel disease risk
Source: Evol Med Public Health. 2026 Jun 11;14(1):1–12. doi: 10.1093/emph/eoag011 (PMC13356811; doi:10.1093/emph/eoag011)
Supplement: Supplementary_material_eoag011 [file supplementary_material_eoag011.zip › Table S3.pdf]

Table S3. Accompanying values for Fig. 2 including relative risks (RR), absolute risk difference (ARD), number needed to treat (NNT(B/H) or benefit/harm).

		appendectomy				
Disease		RR	ER	ARD	NNT(B/H)	CR
infectious	all	1.21(1.12-1.30)	14.61	+2.54 %	39 (H)	12.07
allergic	all	1.23(1.11-1.37)	3.43	+0.65 %	153 (H)	2.78
	urticaria/angiodema	1.38(1.15-1.67)	0.94	+0.27 %	377 (H)	0.68
skin	all	1.27(1.20-1.34)	7.95	+1.70 %	59 (H)	6.25
respiratory	all	1.24(1.17-1.30)	25.08	+4.89 %	20 (H)	20.19
	upper	1.20(1.13-1.27)	12.96	+2.19 %	46 (H)	10.77
	lower	1.39(1.19-1.61)	11.50	+3.24 %	31 (H)	8.26
	chronic lower	1.28(1.17-1.41)	7.84	+1.76 %	57 (H)	6.08
	asthma	1.29(1.17-1.42)	7.50	+1.70 %	59 (H)	5.80
	influenza	1.73(1.39-2.16)	0.73	+0.31 %	319 (H)	0.42
	pneumonia	1.42(1.21-1.66)	7.67	+2.28 %	44 (H)	5.40
digestive	all	1.47(1.26-1.71)	8.74	+2.81 %	36 (H)	5.93
	IBD	0.58(0.44-0.75)	0.14	-0.11 %	-926 (B)	0.25
	liver	1.51(1.03-2.20)	0.12	+0.04 %	2449 (H)	0.07
	pancreatitis	1.64(1.12-2.42)	0.06	+0.03 %	3805 (H)	0.04
	ulcer	1.45(1.11-1.90)	0.14	+0.05 %	2207 (H)	0.09
endocrine	all	1.12(1.04-1.21)	3.09	+0.34 %	294 (H)	2.75
genitourinary	all	1.30(1.20-1.41)	3.73	+0.88 %	114 (H)	2.85
	kidney infection	1.72(1.44-2.06)	1.38	+0.58 %	171 (H)	0.80
musculoskeletal	all	1.21(1.17-1.26)	10.91	+1.96 %	51 (H)	8.95
neoplasms	all	1.10(1.02-1.19)	2.99	+0.29 %	339 (H)	2.70
	benign	1.15(1.06-1.25)	2.64	+0.36 %	276 (H)	2.28
circulatory	all	1.10(0.99-1.24)	-	-	-	1.12
nervous	all	1.22(1.07-1.40)	1.09	+0.20 %	491 (H)	0.89
mental	all	1.22(1.16-1.29)	3.55	+0.66 %	152 (H)	2.89

Footnotes: Numbers are presented only for analyses with sufficient power for hypothesis testing (see methods)

CR - Control Risk (i.e. event rate in the control group)

ER - Experimental Risk (i.e. event rate in the experimental (surgery) group)

ARD - Absolute Risk Difference (a.k.a absolute risk reduction/increase) [ARD=100 X CR X (1-RR)

NNT(B/H) - Number Needed to Treat-Benefit or Harm [NNT=100/ARD]

ER/ARD/NNT only presented for those with a significat RR P-value
