# Supplementary material for: Childhood appendectomy is linked with higher digestive, respiratory, and genitourinary disease risk but lower inflammatory bowel disease risk
Source: Evol Med Public Health. 2026 Jun 11;14(1):1–12. doi: 10.1093/emph/eoag011 (PMC13356811; doi:10.1093/emph/eoag011)
Supplement: Supplementary_material_eoag011 [file supplementary_material_eoag011.zip › Table S4.pdf]

Table S4 - Full Cox regression outputs - association of appendectomy with later disease risk.

Infectious - all	exp(coef)	LCL	UCL	SE	Z	Pr(Z)
surgery	1.2104	1.1250	1.3024	0.0373	5.1139	<0.0001
paternal age	0.9942	0.9789	1.0097	0.0078	-0.7344	0.4626
maternal age	0.9732	0.9561	0.9906	0.0090	-2.9965	0.0027
gestation length	0.9943	0.9819	1.0068	0.0063	-0.8875	0.3747
maternal bleeding	1.0888	1.0430	1.1367	0.0219	3.8783	0.0001
fetal oxygen deprivation	0.8689	0.6649	1.1355	0.1365	-1.0288	0.3035
pregnancy oedema	1.0516	0.9567	1.1560	0.0482	1.0438	0.2965
apgar5 score	1.0342	0.9852	1.0857	0.0247	1.3590	0.1741
birth weight	0.9940	0.9825	1.0056	0.0059	-1.0078	0.3135
preexisting hypertension	1.0317	0.7620	1.3968	0.1545	0.2022	0.8397
preexisting diabetes	1.0190	0.8305	1.2502	0.1043	0.1805	0.8567
previous induced abortion	1.0851	1.0544	1.1167	0.0146	5.5816	<0.0001
previous spontaneous abortion	1.0502	1.0178	1.0835	0.0159	3.0706	0.0021
education level	0.9842	0.9712	0.9974	0.0068	-2.3350	0.0195
parental income	0.9911	0.9772	1.0052	0.0072	-1.2323	0.2178
country	1.1128	1.0505	1.1788	0.0293	3.6398	0.0002
region Sjelland	0.8037	0.7756	0.8328	0.0181	-12.0337	<0.0001
region Syddanmark	0.7163	0.6936	0.7397	0.0163	-20.3403	<0.0001
region Midtylland	0.7393	0.7164	0.7630	0.0160	-18.7936	<0.0001
region Nordjylland	0.6321	0.6056	0.6597	0.0217	-21.0420	<0.0001
mother with disorder	1.3378	1.2905	1.3870	0.0183	15.8260	<0.0001
father with disorder	1.2065	1.1617	1.2530	0.0192	9.7315	<0.0001

Allergic - all	exp(coef)	LCL	UCL	SE	Z	Pr(Z)
surgery	1.2338	1.1107	1.3705	0.0536	3.9198	<0.0001
paternal age	1.0092	0.9860	1.0328	0.0118	0.7755	0.4380
maternal age	1.0113	0.9849	1.0385	0.0135	0.8379	0.4020
gestation length	0.9989	0.9807	1.0176	0.0094	-0.1069	0.9148
maternal bleeding	1.1335	1.0651	1.2064	0.0317	3.9459	<0.0001
fetal oxygen deprivation	0.4339	0.2462	0.7648	0.2891	-2.8872	0.0038
pregnancy oedema	1.3069	1.1517	1.4831	0.0645	4.1502	<0.0001
apgar5 score	1.0526	0.9813	1.1290	0.0357	1.4333	0.1517
birth weight	0.9782	0.9614	0.9954	0.0088	-2.4715	0.0134
preexisting hypertension	0.9900	0.6232	1.5726	0.2361	-0.0424	0.9661
preexisting diabetes	0.9292	0.6691	1.2904	0.1675	-0.4381	0.6612
previous induced abortion	1.0581	1.0134	1.1048	0.0220	2.5660	0.0102
previous spontaneous abortion	1.0648	1.0163	1.1155	0.0237	2.6442	0.0081
education level	1.0467	1.0260	1.0679	0.0102	4.4797	<0.0001
parental income	1.0436	1.0218	1.0658	0.0107	3.9690	<0.0001
country	1.3163	1.2093	1.4327	0.0432	6.3554	<0.0001
region Sjelland	0.6923	0.6541	0.7328	0.0289	-12.6813	<0.0001
region Syddanmark	0.9100	0.8692	0.9528	0.0234	-4.0210	<0.0001
region Midtylland	0.7652	0.7297	0.8023	0.0241	-11.0617	<0.0001
region Nordjylland	0.6888	0.6462	0.7342	0.0325	-11.4487	<0.0001
mother with disorder	1.8198	1.6869	1.9631	0.0386	15.4816	<0.0001
father with disorder	1.6397	1.4872	1.8078	0.0498	9.9306	<0.0001

Allergic - urticaria/angiodema	exp(coef)	LCL	UCL	SE	Z	Pr(Z)
surgery	1.3882	1.1528	1.6717	0.0948	3.4607	0.0005
paternal age	1.0215	0.9789	1.0660	0.0217	0.9809	0.3265
maternal age	0.9728	0.9262	1.0217	0.0250	-1.0996	0.2714
gestation length	0.9858	0.9520	1.0208	0.0177	-0.8023	0.4223
maternal bleeding	1.2201	1.0902	1.3655	0.0574	3.4652	0.0005
fetal oxygen deprivation	0.4529	0.1458	1.4068	0.5782	-1.3695	0.1708
pregnancy oedema	1.5654	1.2604	1.9441	0.1105	4.0540	<0.0001
apgar5 score	1.0420	0.9127	1.1897	0.0676	0.6092	0.5423
birth weight	0.9587	0.9278	0.9907	0.0167	-2.5157	0.0118
preexisting hypertension	1.1561	0.5183	2.5788	0.4093	0.3544	0.7230
preexisting diabetes	1.1752	0.6798	2.0317	0.2792	0.5781	0.5631
previous induced abortion	1.0956	1.0118	1.1864	0.0406	2.2508	0.0243
previous spontaneous abortion	1.1072	1.0157	1.2069	0.0439	2.3158	0.0205
education level	1.0127	0.9757	1.0511	0.0189	0.6657	0.5055
parental income	0.9939	0.9559	1.0334	0.0199	-0.3052	0.7601
country	1.4140	1.2275	1.6289	0.0721	4.8007	<0.0001
region Sjelland	0.6071	0.5491	0.6713	0.0512	-9.7291	<0.0001
region Syddanmark	0.6498	0.5966	0.7077	0.0435	-9.8965	<0.0001
region Midtylland	0.4900	0.4467	0.5375	0.0471	-15.1203	<0.0001
region Nordjylland	0.3792	0.3307	0.4347	0.0697	-13.9081	<0.0001
mother with disorder	1.9665	1.5891	2.4335	0.1087	6.2204	<0.0001
father with disorder	1.2273	0.8795	1.7125	0.1699	1.2051	0.2281

Skin - all	exp(coef)	LCL	UCL	SE	Z	Pr(Z)
surgery	1.2721	1.2029	1.3452	0.0285	8.4354	<0.0001
paternal age	0.9864	0.9743	0.9986	0.0062	-2.1794	0.0293
maternal age	0.9539	0.9405	0.9675	0.0072	-6.5290	<0.0001
gestation length	0.9982	0.9884	1.0082	0.0050	-0.3361	0.7367
maternal bleeding	1.0620	1.0262	1.0991	0.0174	3.4441	0.0005
fetal oxygen deprivation	0.8065	0.6524	0.9970	0.1081	-1.9868	0.0469
pregnancy oedema	1.1265	1.0491	1.2097	0.0363	3.2810	0.0010
apgar5 score	1.1068	1.0665	1.1487	0.0189	5.3671	<0.0001
birth weight	1.0297	1.0201	1.0393	0.0047	6.1577	<0.0001
preexisting hypertension	1.2039	0.9610	1.5083	0.1149	1.6144	0.1064
preexisting diabetes	1.2145	1.0494	1.4056	0.0745	2.6076	0.0091
previous induced abortion	1.0907	1.0659	1.1162	0.0117	7.3966	<0.0001
previous spontaneous abortion	1.0579	1.0316	1.0849	0.0128	4.3795	<0.0001
education level	0.9549	0.9448	0.9652	0.0054	-8.4429	<0.0001
parental income	0.9734	0.9624	0.9846	0.0058	-4.6270	<0.0001
country	1.2484	1.1949	1.3043	0.0223	9.9270	<0.0001
region Sjelland	0.8174	0.7936	0.8418	0.0150	-13.3998	<0.0001
region Syddanmark	0.8796	0.8575	0.9022	0.0129	-9.8965	<0.0001
region Midtylland	0.8754	0.8537	0.8977	0.0128	-10.3730	<0.0001
region Nordjylland	0.6199	0.5979	0.6427	0.0184	-25.9748	<0.0001
mother with disorder	1.3242	1.2899	1.3594	0.0133	20.9786	<0.0001
father with disorder	1.2716	1.2376	1.3066	0.0138	17.3785	<0.0001

Respiratory - all	exp(coef)	LCL	UCL	SE	Z	Pr(Z)
surgery	1.2421	1.1784	1.3093	0.0268	8.0682	<0.0001
paternal age	0.9831	0.9721	0.9943	0.0057	-2.9380	0.0033
maternal age	0.9295	0.9174	0.9417	0.0066	-10.9950	<0.0001
gestation length	1.0088	0.9996	1.0181	0.0046	1.8842	0.0595
maternal bleeding	1.0829	1.0487	1.1181	0.0163	4.8761	<0.0001
fetal oxygen deprivation	0.7861	0.6411	0.9638	0.1039	-2.3143	0.0206
pregnancy oedema	1.1364	1.0625	1.2155	0.0343	3.7271	0.0001
apgar5 score	1.0663	1.0291	1.1048	0.0180	3.5507	0.0003
birth weight	1.0067	0.9982	1.0153	0.0043	1.5566	0.1195
preexisting hypertension	1.0680	0.8574	1.3302	0.1120	0.5873	0.5569
preexisting diabetes	1.0346	0.8893	1.2035	0.0771	0.4408	0.6593
previous induced abortion	1.0846	1.0619	1.1078	0.0107	7.5369	<0.0001
previous spontaneous abortion	1.0399	1.0162	1.0641	0.0117	3.3329	0.0008
education level	0.9678	0.9584	0.9773	0.0049	-6.5691	<0.0001
parental income	0.9819	0.9717	0.9922	0.0053	-3.4294	0.0006
country	0.9648	0.9231	1.0084	0.0225	-1.5857	0.1127
region Sjelland	0.9109	0.8871	0.9353	0.0134	-6.9142	<0.0001
region Syddanmark	0.8372	0.8176	0.8573	0.0120	-14.6868	<0.0001
region Midtylland	0.8282	0.8091	0.8478	0.0118	-15.8379	<0.0001
region Nordjylland	0.8367	0.8123	0.8617	0.0150	-11.8375	<0.0001
mother with disorder	1.3427	1.3157	1.3703	0.0103	28.4547	<0.0001
father with disorder	1.2404	1.2152	1.2660	0.0104	20.6143	<0.0001

Respiratory - upper	exp(coef)	LCL	UCL	SE	Z	Pr(Z)
surgery	1.2032	1.1354	1.2752	0.0296	6.2469	<0.0001
paternal age	0.9828	0.9706	0.9952	0.0063	-2.7119	0.0066
maternal age	0.9315	0.9182	0.9450	0.0073	-9.6555	<0.0001
gestation length	1.0151	1.0050	1.0253	0.0051	2.9469	0.0032
maternal bleeding	1.0807	1.0437	1.1191	0.0178	4.3616	<0.0001
fetal oxygen deprivation	0.6940	0.5468	0.8807	0.1215	-3.0042	0.0026
pregnancy oedema	1.1779	1.0949	1.2672	0.0372	4.3942	<0.0001
apgar5 score	1.1019	1.0607	1.1447	0.0194	4.9961	<0.0001
birth weight	1.0079	0.9985	1.0174	0.0047	1.6619	0.0965
preexisting hypertension	0.9964	0.7780	1.2760	0.1262	-0.0282	0.9774
preexisting diabetes	1.0538	0.8946	1.2413	0.0835	0.6272	0.5304
previous induced abortion	1.1080	1.0827	1.1338	0.0117	8.7255	<0.0001
previous spontaneous abortion	1.0454	1.0194	1.0721	0.0128	3.4535	0.0005
education level	0.9653	0.9549	0.9757	0.0054	-6.4277	<0.0001
parental income	0.9904	0.9791	1.0018	0.0058	-1.6487	0.0991
country	0.9367	0.8917	0.9840	0.0251	-2.5990	0.0093
region Sjelland	0.9450	0.9179	0.9729	0.0148	-3.8112	0.0001
region Syddanmark	0.8635	0.8412	0.8864	0.0133	-10.9933	<0.0001
region Midtylland	0.8755	0.8533	0.8982	0.0131	-10.1461	<0.0001
region Nordjylland	0.8783	0.8503	0.9073	0.0165	-7.8325	<0.0001
mother with disorder	1.4703	1.4317	1.5099	0.0135	28.4228	<0.0001
father with disorder	1.3051	1.2688	1.3425	0.0144	18.4885	<0.0001

Respiratory - lower	exp(coef)	LCL	UCL	SE	Z	Pr(Z)
**surgery**	1.3917	1.1961	1.6194	0.0772	4.2777	<0.0001
**paternal age**	0.9942	0.9606	1.0290	0.0175	-0.3258	0.7445
**maternal age**	0.8993	0.8641	0.9360	0.0203	-5.2030	<0.0001
**gestation length**	0.9496	0.9230	0.9769	0.0144	-3.5673	0.0003
**maternal bleeding**	1.1373	1.0346	1.2503	0.0482	2.6656	0.0076
**fetal oxygen deprivation**	1.0837	0.6279	1.8705	0.2784	0.2890	0.7725
**pregnancy oedema**	1.1295	0.9221	1.3835	0.1035	1.1766	0.2393
**apgar5 score**	1.1163	1.0044	1.2407	0.0538	2.0424	0.0411
**birth weight**	0.9770	0.9514	1.0033	0.0135	-1.7160	0.0861
**preexisting hypertension**	0.8939	0.4255	1.8782	0.3787	-0.2958	0.7673
**preexisting diabetes**	1.3016	0.8693	1.9490	0.2059	1.2801	0.2005
**previous induced abortion**	1.0179	0.9523	1.0880	0.0339	0.5221	0.6015
**previous spontaneous abortion**	1.1201	1.0438	1.2019	0.0359	3.1528	0.0016
**education level**	0.9611	0.9324	0.9907	0.0154	-2.5607	0.0104
**parental income**	0.8992	0.8707	0.9286	0.0164	-6.4630	<0.0001
**country**	1.0521	0.9304	1.1898	0.0627	0.8106	0.4175
**region Sjelland**	0.7682	0.7083	0.8331	0.0414	-6.3678	<0.0001
**region Syddanmark**	0.6988	0.6498	0.7516	0.0371	-9.6535	<0.0001
**region Midtylland**	0.6495	0.6035	0.6991	0.0374	-11.5050	<0.0001
**region Nordjylland**	0.6699	0.6101	0.7356	0.0477	-8.3959	<0.0001
**mother with disorder**	1.6437	1.4852	1.8191	0.0517	9.6081	<0.0001
**father with disorder**	1.3244	1.1953	1.4674	0.0523	5.3707	<0.0001

Respiratory - chronic lower	exp(coef)	LCL	UCL	SE	Z	Pr(Z)
**surgery**	1.2891	1.1720	1.4180	0.0486	5.2254	<0.0001
**paternal age**	0.9896	0.9692	1.0104	0.0106	-0.9791	0.3275
**maternal age**	0.9490	0.9263	0.9722	0.0123	-4.2417	<0.0001
**gestation length**	0.9781	0.9617	0.9948	0.0086	-2.5524	0.0106
**maternal bleeding**	1.1149	1.0527	1.1807	0.0292	3.7178	0.0002
**fetal oxygen deprivation**	0.9896	0.6869	1.4259	0.1863	-0.0555	0.9556
**pregnancy oedema**	1.1343	0.9998	1.2869	0.0643	1.9582	0.0501
**apgar5 score**	1.0445	0.9793	1.1139	0.0328	1.3259	0.1848
**birth weight**	0.9742	0.9588	0.9899	0.0081	-3.1938	0.0014
**preexisting hypertension**	0.8541	0.5444	1.3399	0.2297	-0.6862	0.4925
**preexisting diabetes**	0.9875	0.7450	1.3089	0.1437	-0.0871	0.9305
**previous induced abortion**	1.0400	0.9996	1.0820	0.0202	1.9410	0.0522
**previous spontaneous abortion**	1.0708	1.0259	1.1176	0.0218	3.1308	0.0017
**education level**	1.0046	0.9861	1.0234	0.0094	0.4881	0.6254
**parental income**	0.9359	0.9177	0.9544	0.0100	-6.6108	<0.0001
**country**	0.8692	0.8010	0.9433	0.0417	-3.3565	0.0007
**region Sjelland**	0.7914	0.7529	0.8318	0.0254	-9.1994	<0.0001
**region Syddanmark**	0.8511	0.8153	0.8886	0.0219	-7.3375	<0.0001
**region Midtylland**	0.6981	0.6674	0.7302	0.0229	-15.6718	<0.0001
**region Nordjylland**	0.8055	0.7625	0.8510	0.0279	-7.7243	<0.0001
**mother with disorder**	1.9157	1.8141	2.0231	0.0278	23.3679	<0.0001
**father with disorder**	1.8138	1.7083	1.9258	0.0305	19.4768	<0.0001

Respiratory - asthma	exp(coef)	LCL	UCL	SE	Z	Pr(Z)
surgery	1.2924	1.1739	1.4229	0.0490	5.2286	<0.0001
paternal age	1.0001	0.9793	1.0214	0.0107	0.0144	0.9884
maternal age	0.9499	0.9270	0.9734	0.0124	-4.1138	<0.0001
gestation length	0.9747	0.9582	0.9915	0.0087	-2.9332	0.0033
maternal bleeding	1.1224	1.0595	1.1891	0.0294	3.9246	<0.0001
fetal oxygen deprivation	1.0082	0.6997	1.4526	0.1863	0.0440	0.9648
pregnancy oedema	1.1235	0.9883	1.2772	0.0654	1.7806	0.0749
apgar5 score	1.0466	0.9809	1.1167	0.0330	1.3791	0.1678
birth weight	0.9727	0.9571	0.9885	0.0082	-3.3550	0.0007
preexisting hypertension	0.8636	0.5505	1.3550	0.2297	-0.6378	0.5235
preexisting diabetes	0.9634	0.7226	1.2844	0.1467	-0.2538	0.7996
previous induced abortion	1.0449	1.0039	1.0875	0.0203	2.1534	0.0312
previous spontaneous abortion	1.0743	1.0288	1.1217	0.0220	3.2502	0.0011
education level	0.9951	0.9767	1.0139	0.0095	-0.5045	0.6138
parental income	0.9320	0.9138	0.9507	0.0100	-6.9650	<0.0001
country	0.8443	0.7771	0.9173	0.0422	-4.0001	<0.0001
region Sjelland	0.7905	0.7516	0.8313	0.0256	-9.1537	<0.0001
region Syddanmark	0.8478	0.8117	0.8854	0.0221	-7.4455	<0.0001
region Midtylland	0.7009	0.6698	0.7333	0.0230	-15.3853	<0.0001
region Nordjylland	0.7982	0.7551	0.8437	0.0283	-7.9610	<0.0001
mother with disorder	2.1451	2.0193	2.2786	0.0308	24.7669	<0.0001
father with disorder	2.1221	1.9720	2.2837	0.0374	20.1031	<0.0001

Respiratory - influenza	exp(coef)	LCL	UCL	SE	Z	Pr(Z)
surgery	1.7354	1.3933	2.1615	0.1120	4.9207	<0.0001
paternal age	1.0166	0.9640	1.0720	0.0271	0.6082	0.5430
maternal age	0.8843	0.8309	0.9413	0.0318	-3.8607	0.0001
gestation length	0.9192	0.8790	0.9612	0.0228	-3.6922	0.0002
maternal bleeding	1.1167	0.9621	1.2962	0.0760	1.4522	0.1464
fetal oxygen deprivation	1.8324	0.8697	3.8604	0.3801	1.5930	0.1111
pregnancy oedema	1.3593	0.9958	1.8553	0.1587	1.9339	0.0531
apgar5 score	1.0448	0.8829	1.2364	0.0858	0.5109	0.6094
birth weight	0.9700	0.9298	1.0120	0.0216	-1.4074	0.1592
preexisting hypertension	3.1256	1.6727	5.8406	0.3189	3.5727	0.0003
preexisting diabetes	1.4147	0.7784	2.5714	0.3048	1.1382	0.2550
previous induced abortion	1.0961	0.9889	1.2150	0.0525	1.7484	0.0803
previous spontaneous abortion	1.0841	0.9683	1.2137	0.0576	1.4017	0.1609
education level	0.9508	0.9068	0.9969	0.0241	-2.0857	0.0370
parental income	0.9164	0.8709	0.9642	0.0259	-3.3633	0.0007
country	2.0670	1.7840	2.3949	0.0751	9.6658	<0.0001
region Sjelland	0.6671	0.5877	0.7571	0.0646	-6.2659	<0.0001
region Syddanmark	0.5681	0.5060	0.6379	0.0591	-9.5624	<0.0001
region Midtylland	0.4954	0.4393	0.5587	0.0613	-11.4447	<0.0001
region Nordjylland	0.4667	0.3959	0.5502	0.0839	-9.0744	<0.0001
mother with disorder	2.3602	1.7678	3.1510	0.1474	5.8244	<0.0001
father with disorder	1.7513	1.1790	2.6015	0.2018	2.7757	0.0055

Respiratory - pneumonia	exp(coef)	LCL	UCL	SE	Z	Pr(Z)
surgery	1.4213	1.2141	1.6639	0.0803	4.3737	<0.0001
paternal age	0.9885	0.9534	1.0249	0.0184	-0.6221	0.5338
maternal age	0.9036	0.8664	0.9423	0.0214	-4.7281	<0.0001
gestation length	0.9460	0.9183	0.9746	0.0151	-3.6503	0.0002
maternal bleeding	1.1856	1.0756	1.3069	0.0496	3.4270	0.0006
fetal oxygen deprivation	0.9396	0.5044	1.7500	0.3173	-0.1963	0.8443
pregnancy oedema	1.1804	0.9572	1.4558	0.1069	1.5511	0.1208
apgar5 score	1.1535	1.0346	1.2861	0.0555	2.5739	0.0100
birth weight	0.9805	0.9536	1.0083	0.0142	-1.3784	0.1680
preexisting hypertension	0.9971	0.4745	2.0952	0.3788	-0.0075	0.9939
preexisting diabetes	1.2239	0.7949	1.8842	0.2201	0.9178	0.3587
previous induced abortion	1.0177	0.9491	1.0913	0.0356	0.4934	0.6217
previous spontaneous abortion	1.0916	1.0132	1.1761	0.0380	2.3057	0.0211
education level	0.9589	0.9288	0.9901	0.0162	-2.5693	0.0101
parental income	0.9112	0.8809	0.9426	0.0172	-5.3776	<0.0001
country	1.0595	0.9320	1.2044	0.0653	0.8846	0.3763
region Sjelland	0.7380	0.6777	0.8036	0.0434	-6.9888	<0.0001
region Syddanmark	0.6902	0.6399	0.7445	0.0386	-9.6028	<0.0001
region Midtylland	0.6135	0.5677	0.6631	0.0396	-12.3251	<0.0001
region Nordjylland	0.6126	0.5541	0.6773	0.0512	-9.5671	<0.0001
mother with disorder	1.6450	1.4711	1.8395	0.0570	8.7323	<0.0001
father with disorder	1.3344	1.1939	1.4913	0.0567	5.0843	<0.0001

Digestive - all	exp(coef)	LCL	UCL	SE	Z	Pr(Z)
surgery	1.4730	1.2638	1.7168	0.0781	4.9572	<0.0001
paternal age	0.9720	0.9584	0.9858	0.0071	-3.9338	<0.0001
maternal age	0.9010	0.8864	0.9159	0.0083	-12.4854	<0.0001
gestation length	0.9729	0.9618	0.9842	0.0058	-4.6675	<0.0001
maternal bleeding	1.0449	1.0040	1.0875	0.0203	2.1591	0.0308
fetal oxygen deprivation	1.0296	0.8274	1.2812	0.1115	0.2621	0.7932
pregnancy oedema	1.2218	1.1311	1.3197	0.0393	5.0928	<0.0001
apgar5 score	1.0620	1.0164	1.1096	0.0223	2.6879	0.0071
birth weight	0.9538	0.9437	0.9641	0.0054	-8.6175	<0.0001
preexisting hypertension	1.0242	0.7690	1.3639	0.1461	0.1636	0.8700
preexisting diabetes	1.2137	1.0172	1.4481	0.0900	2.1502	0.0315
previous induced abortion	1.0276	1.0000	1.0559	0.0138	1.9660	0.0492
previous spontaneous abortion	1.0494	1.0192	1.0805	0.0148	3.2424	0.0011
education level	0.9170	0.9058	0.9284	0.0062	-13.7408	<0.0001
parental income	0.9508	0.9384	0.9635	0.0067	-7.4893	<0.0001
country	0.8930	0.8449	0.9439	0.0282	-3.9987	<0.0001
region Sjelland	0.9645	0.9323	0.9977	0.0173	-2.0884	0.0367
region Syddanmark	1.0421	1.0118	1.0733	0.0150	2.7449	0.0060
region Midtylland	0.8717	0.8454	0.8987	0.0156	-8.8007	<0.0001
region Nordjylland	0.9344	0.9001	0.9700	0.0190	-3.5519	0.0003
mother with disorder	1.3181	1.2882	1.3486	0.0116	23.6514	<0.0001
father with disorder	1.2333	1.2056	1.2616	0.0115	18.0951	<0.0001

Digestive - inflammatory bowel disease	exp(coef)	LCL	UCL	SE	Z	Pr(Z)
surgery	0.5807	0.4439	0.7596	0.1370	-3.9660	<0.0001
paternal age	0.9982	0.9568	1.0415	0.0216	-0.0790	0.9369
maternal age	0.9728	0.9268	1.0212	0.0247	-1.1102	0.2668
gestation length	0.9792	0.9468	1.0127	0.0171	-1.2204	0.2223
maternal bleeding	1.0406	0.9259	1.1696	0.0596	0.6689	0.5035
fetal oxygen deprivation	1.5195	0.8971	2.5736	0.2688	1.5564	0.1196
pregnancy oedema	1.2880	1.0322	1.6073	0.1129	2.2411	0.0250
apgar5 score	0.9563	0.8361	1.0939	0.0685	-0.6505	0.5153
birth weight	0.9954	0.9646	1.0272	0.0160	-0.2842	0.7762
preexisting hypertension	1.4974	0.7475	2.9995	0.3544	1.1391	0.2546
preexisting diabetes	1.1513	0.6794	1.9509	0.2690	0.5237	0.6004
previous induced abortion	0.9634	0.8881	1.0451	0.0415	-0.8957	0.3703
previous spontaneous abortion	1.0978	1.0095	1.1938	0.0427	2.1823	0.0290
education level	0.9724	0.9377	1.0084	0.0185	-1.5072	0.1317
parental income	1.0465	1.0069	1.0877	0.0197	2.3090	0.0209
country	0.8608	0.7132	1.0391	0.0960	-1.5602	0.1187
region Sjelland	1.2798	1.1632	1.4080	0.0487	5.0645	<0.0001
region Syddanmark	1.1194	1.0244	1.2231	0.0452	2.4943	0.0126
region Midtylland	0.9764	0.8918	1.0691	0.0462	-0.5144	0.6069
region Nordjylland	1.0010	0.8944	1.1202	0.0574	0.0174	0.9860
mother with disorder	3.5528	3.0269	4.1701	0.0817	15.5111	<0.0001
father with disorder	3.5168	2.9406	4.2059	0.0912	13.7746	<0.0001

Digestive - liver	exp(coef)	LCL	UCL	SE	Z	Pr(Z)
surgery	1.5129	1.0375	2.2061	0.1924	2.1517	0.0314
paternal age	0.9776	0.8934	1.0697	0.0459	-0.4925	0.6222
maternal age	0.8917	0.8032	0.9900	0.0533	-2.1471	0.0317
gestation length	0.9630	0.8948	1.0365	0.0374	-1.0033	0.3157
maternal bleeding	1.0489	0.8141	1.3515	0.1293	0.3697	0.7115
fetal oxygen deprivation	1.0078	0.2506	4.0530	0.7100	0.0110	0.9911
pregnancy oedema	1.0909	0.6412	1.8558	0.2710	0.3210	0.7481
apgar5 score	1.4633	1.1433	1.8730	0.1259	3.0236	0.0024
birth weight	0.9911	0.9248	1.0621	0.0353	-0.2525	0.8006
preexisting hypertension	0.9011	0.1265	6.4176	1.0016	-0.1039	0.9172
preexisting diabetes	0.3680	0.0516	2.6254	1.0024	-0.9970	0.3187
previous induced abortion	0.8908	0.7422	1.0692	0.0931	-1.2411	0.2145
previous spontaneous abortion	1.2560	1.0503	1.5020	0.0912	2.4985	0.0124
education level	0.9051	0.8362	0.9796	0.0403	-2.4683	0.0135
parental income	0.9439	0.8675	1.0271	0.0430	-1.3378	0.1809
country	1.3063	0.9712	1.7570	0.1512	1.7672	0.0771
region Sjelland	0.9926	0.8102	1.2161	0.1035	-0.0708	0.9434
region Syddanmark	0.7107	0.5834	0.8657	0.1006	-3.3924	0.0006
region Midtylland	0.8084	0.6691	0.9768	0.0965	-2.2022	0.0276
region Nordjylland	0.6338	0.4878	0.8235	0.1335	-3.4135	0.0006
mother with disorder	2.1841	1.4117	3.3792	0.2226	3.5085	0.0004
father with disorder	1.6064	1.0282	2.5098	0.2276	2.0822	0.0373

Digestive - pancreatitis	exp(coef)	LCL	UCL	SE	Z	Pr(Z)
surgery	1.6485	1.1215	2.4230	0.1965	2.5438	0.0109
paternal age	0.9278	0.8407	1.0238	0.0502	-1.4901	0.1361
maternal age	0.9354	0.8351	1.0478	0.0578	-1.1525	0.2490
gestation length	1.0031	0.9247	1.0880	0.0414	0.0747	0.9403
maternal bleeding	1.0570	0.7977	1.4008	0.1436	0.3865	0.6991
fetal oxygen deprivation	0.0000	0.0000	Inf	835.2352	-0.0168	0.9865
pregnancy oedema	1.1328	0.6652	1.9291	0.2715	0.4594	0.6459
apgar5 score	1.1385	0.8398	1.5434	0.1552	0.8358	0.4032
birth weight	0.9566	0.8869	1.0317	0.0385	-1.1486	0.2507
preexisting hypertension	0.0000	0.0000	Inf	1154.7723	-0.0121	0.9903
preexisting diabetes	2.0413	0.7579	5.4975	0.5054	1.4117	0.1580
previous induced abortion	1.0440	0.8632	1.2627	0.0970	0.4443	0.6567
previous spontaneous abortion	0.9353	0.7550	1.1587	0.1092	-0.6115	0.5408
education level	0.8316	0.7622	0.9072	0.0444	-4.1492	<0.0001
parental income	0.8198	0.7477	0.8988	0.0469	-4.2319	<0.0001
country	0.3872	0.2313	0.6483	0.2629	-3.6079	0.0003
region Sjelland	0.8651	0.6766	1.1062	0.1253	-1.1549	0.2481
region Syddanmark	0.9997	0.8120	1.2307	0.1060	-0.0024	0.9980
region Midtylland	0.8926	0.7207	1.1056	0.1091	-1.0399	0.2983
region Nordjylland	0.8730	0.6690	1.1392	0.1357	-0.9998	0.3173
mother with disorder	1.8820	0.9732	3.6394	0.3364	1.8794	0.0601
father with disorder	1.5141	0.9048	2.5338	0.2626	1.5792	0.1142

Digestive - ulcerative	exp(coef)	LCL	UCL	SE	Z	Pr(Z)
surgery	1.4588	1.1146	1.9094	0.1373	2.7504	0.0059
paternal age	1.0208	0.9590	1.0866	0.0318	0.6464	0.5179
maternal age	0.8268	0.7680	0.8901	0.0376	-5.0536	<0.0001
gestation length	0.9919	0.9408	1.0456	0.0269	-0.3017	0.7628
maternal bleeding	1.0257	0.8536	1.2326	0.0937	0.2717	0.7858
fetal oxygen deprivation	0.5231	0.1304	2.0976	0.7085	-0.9144	0.3604
pregnancy oedema	1.3545	0.9756	1.8806	0.1674	1.8127	0.0698
apgar5 score	1.2166	1.0035	1.4749	0.0982	1.9961	0.0459
birth weight	0.9441	0.8987	0.9917	0.0251	-2.2892	0.0220
preexisting hypertension	1.4092	0.4530	4.3838	0.5790	0.5925	0.5534
preexisting diabetes	1.7968	0.9279	3.4793	0.3371	1.7381	0.0821
previous induced abortion	1.0512	0.9280	1.1908	0.0635	0.7864	0.4316
previous spontaneous abortion	1.0330	0.9020	1.1829	0.0691	0.4698	0.6384
education level	0.8356	0.7898	0.8841	0.0287	-6.2415	<0.0001
parental income	0.8763	0.8248	0.9310	0.0308	-4.2736	<0.0001
country	1.1448	0.9191	1.4260	0.1120	1.2071	0.2273
region Sjelland	1.1563	0.9952	1.3434	0.0765	1.8981	0.0576
region Syddanmark	0.8750	0.7575	1.0106	0.0735	-1.8159	0.0693
region Midtylland	0.8445	0.7303	0.9765	0.0741	-2.2797	0.0226
region Nordjylland	1.2699	1.0831	1.4889	0.0811	2.9436	0.0032
mother with disorder	1.7602	1.3930	2.2243	0.1193	4.7362	<0.0001
father with disorder	1.6635	1.3717	2.0174	0.0984	5.1715	<0.0001

Endocrine - all	exp(coef)	LCL	UCL	SE	Z	Pr(Z)
surgery	1.1232	1.0401	1.2129	0.0392	2.9643	0.0030
paternal age	0.9448	0.9294	0.9604	0.0083	-6.7730	<0.0001
maternal age	0.8523	0.8361	0.8687	0.0097	-16.3819	<0.0001
gestation length	0.9943	0.9808	1.0079	0.0069	-0.8176	0.4135
maternal bleeding	1.0802	1.0305	1.1323	0.0240	3.2116	0.0013
fetal oxygen deprivation	1.1271	0.8812	1.4417	0.1255	0.9534	0.3403
pregnancy oedema	1.2692	1.1695	1.3773	0.0417	5.7152	<0.0001
apgar5 score	1.1587	1.1013	1.2190	0.0258	5.6886	<0.0001
birth weight	1.0373	1.0243	1.0505	0.0064	5.6822	<0.0001
preexisting hypertension	1.0739	0.7774	1.4835	0.1648	0.4325	0.6653
preexisting diabetes	1.4498	1.2200	1.7228	0.0880	4.2199	<0.0001
previous induced abortion	1.0099	0.9777	1.0433	0.0165	0.6004	0.5482
previous spontaneous abortion	1.0403	1.0047	1.0772	0.0177	2.2265	0.0259
education level	0.8490	0.8367	0.8614	0.0074	-22.0802	<0.0001
parental income	0.8787	0.8651	0.8925	0.0079	-16.2953	<0.0001
country	0.8077	0.7574	0.8614	0.0328	-6.5036	<0.0001
region Sjelland	1.2548	1.2072	1.3043	0.0197	11.4981	<0.0001
region Syddanmark	1.2248	1.1828	1.2683	0.0177	11.3935	<0.0001
region Midtylland	0.8504	0.8188	0.8832	0.0193	-8.3836	<0.0001
region Nordjylland	0.9178	0.8764	0.9612	0.0235	-3.6353	0.0002
mother with disorder	1.6355	1.5881	1.6842	0.0149	32.8370	<0.0001
father with disorder	1.5009	1.4505	1.5531	0.0174	23.3052	<0.0001

Genitourinary - all	exp(coef)	LCL	UCL	SE	Z	Pr(Z)
surgery	1.3070	1.2073	1.4148	0.0404	6.6188	<0.0001
paternal age	0.9565	0.9398	0.9735	0.0090	-4.9316	<0.0001
maternal age	0.8897	0.8715	0.9082	0.0105	-11.1003	<0.0001
gestation length	0.9875	0.9732	1.0020	0.0074	-1.6902	0.0909
maternal bleeding	1.1044	1.0515	1.1600	0.0250	3.9657	<0.0001
fetal oxygen deprivation	0.8454	0.6092	1.1732	0.1671	-1.0042	0.3152
pregnancy oedema	1.1567	1.0441	1.2814	0.0522	2.7861	0.0053
apgar5 score	1.0351	0.9782	1.0953	0.0288	1.1978	0.2309
birth weight	0.9557	0.9427	0.9689	0.0069	-6.4834	<0.0001
preexisting hypertension	1.0258	0.7209	1.4597	0.1799	0.1416	0.8873
preexisting diabetes	1.1100	0.8811	1.3985	0.1178	0.8860	0.3755
previous induced abortion	1.1287	1.0921	1.1665	0.0168	7.2024	<0.0001
previous spontaneous abortion	1.0689	1.0303	1.1089	0.0187	3.5518	0.0003
education level	0.9215	0.9073	0.9361	0.0079	-10.2398	<0.0001
parental income	0.9187	0.9037	0.9339	0.0083	-10.1066	<0.0001
country	0.8213	0.7683	0.8781	0.0340	-5.7707	<0.0001
region Sjelland	0.6922	0.6640	0.7215	0.0211	-17.3685	<0.0001
region Syddanmark	0.6407	0.6174	0.6648	0.0188	-23.5769	<0.0001
region Midtylland	0.5909	0.5691	0.6136	0.0192	-27.3775	<0.0001
region Nordjylland	0.5911	0.5631	0.6205	0.0247	-21.2193	<0.0001
mother with disorder	1.3147	1.2713	1.3596	0.0171	15.9717	<0.0001
father with disorder	1.2770	1.2184	1.3385	0.0239	10.1989	<0.0001

Genitourinary - kid.infec	exp(coef)	LCL	UCL	SE	Z	Pr(Z)
surgery	1.7243	1.4407	2.0637	0.0916	5.9437	<0.0001
paternal age	0.9945	0.9503	1.0406	0.0231	-0.2381	0.8117
maternal age	0.8479	0.8037	0.8945	0.0272	-6.0410	<0.0001
gestation length	1.0169	0.9797	1.0555	0.0190	0.8829	0.3772
maternal bleeding	1.1338	1.0003	1.2850	0.0638	1.9659	0.0492
fetal oxygen deprivation	0.8167	0.3391	1.9669	0.4484	-0.4514	0.6516
pregnancy oedema	0.9877	0.7325	1.3318	0.1524	-0.0807	0.9356
apgar5 score	1.1129	0.9661	1.2821	0.0721	1.4822	0.1382
birth weight	0.9832	0.9493	1.0184	0.0179	-0.9416	0.3463
preexisting hypertension	0.8272	0.3098	2.2082	0.5009	-0.3786	0.7049
preexisting diabetes	1.4834	0.9041	2.4339	0.2526	1.5610	0.1185
previous induced abortion	1.1953	1.1004	1.2983	0.0421	4.2295	<0.0001
previous spontaneous abortion	1.1387	1.0385	1.2486	0.0470	2.7637	0.0057
education level	0.9395	0.9020	0.9785	0.0207	-3.0059	0.0026
parental income	0.9383	0.8992	0.9791	0.0217	-2.9292	0.0033
country	0.5220	0.4240	0.6426	0.1060	-6.1290	<0.0001
region Sjelland	0.6414	0.5751	0.7154	0.0556	-7.9737	<0.0001
region Syddanmark	0.6029	0.5473	0.6642	0.0493	-10.2430	<0.0001
region Midtylland	0.6356	0.5783	0.6986	0.0482	-9.4005	<0.0001
region Nordjylland	0.6618	0.5875	0.7455	0.0607	-6.7921	<0.0001
mother with disorder	1.6177	1.2977	2.0164	0.1124	4.2785	<0.0001
father with disorder	1.6399	1.0440	2.5758	0.2303	2.1471	0.0317

Musculoskeletal - all	exp(coef)	LCL	UCL	SE	Z	Pr(Z)
surgery	1.2182	1.1715	1.2668	0.0199	9.8864	<0.0001
paternal age	0.9698	0.9616	0.9781	0.0043	-7.0338	<0.0001
maternal age	0.9309	0.9218	0.9401	0.0050	-14.2524	<0.0001
gestation length	1.0021	0.9952	1.0089	0.0034	0.6041	0.5457
maternal bleeding	1.0770	1.0520	1.1026	0.0119	6.1913	<0.0001
fetal oxygen deprivation	1.1343	1.0048	1.2804	0.0618	2.0388	0.0414
pregnancy oedema	1.0897	1.0354	1.1469	0.0260	3.2980	0.0009
apgar5 score	1.0129	0.9864	1.0401	0.0135	0.9493	0.3424
birth weight	0.9892	0.9829	0.9956	0.0032	-3.3105	0.0009
preexisting hypertension	1.1173	0.9533	1.3096	0.0810	1.3697	0.1707
preexisting diabetes	1.0233	0.9163	1.1428	0.0563	0.4094	0.6822
previous induced abortion	1.0382	1.0217	1.0550	0.0081	4.5811	<0.0001
previous spontaneous abortion	1.0338	1.0160	1.0520	0.0088	3.7545	0.0001
education level	0.9492	0.9422	0.9563	0.0037	-13.7369	<0.0001
parental income	0.9921	0.9842	1.0000	0.0040	-1.9544	0.0506
country	0.8347	0.8066	0.8638	0.0175	-10.3198	<0.0001
region Sjelland	0.8469	0.8294	0.8647	0.0106	-15.6046	<0.0001
region Syddanmark	0.8843	0.8685	0.9004	0.0091	-13.3705	<0.0001
region Midtylland	1.0462	1.0284	1.0643	0.0087	5.1528	<0.0001
region Nordjylland	0.8490	0.8297	0.8687	0.0117	-13.9888	<0.0001
mother with disorder	1.3311	1.3144	1.3481	0.0064	44.2994	<0.0001
father with disorder	1.2327	1.2170	1.2486	0.0065	32.0896	<0.0001

Neoplasms - all	exp(coef)	LCL	UCL	SE	Z	Pr(Z)
surgery	1.1090	1.0254	1.1993	0.0399	2.5899	0.0095
paternal age	0.9876	0.9712	1.0043	0.0085	-1.4512	0.1467
maternal age	0.9658	0.9475	0.9845	0.0097	-3.5560	0.0003
gestation length	1.0037	0.9904	1.0171	0.0067	0.5462	0.5849
maternal bleeding	1.0572	1.0093	1.1073	0.0236	2.3533	0.0186
fetal oxygen deprivation	1.0633	0.8330	1.3573	0.1245	0.4935	0.6216
pregnancy oedema	1.0159	0.9208	1.1209	0.0501	0.3163	0.7517
apgar5 score	1.0252	0.9735	1.0796	0.0263	0.9450	0.3446
birth weight	1.0178	1.0052	1.0305	0.0063	2.7865	0.0053
preexisting hypertension	0.9388	0.6670	1.3212	0.1743	-0.3620	0.7173
preexisting diabetes	0.9604	0.7649	1.2059	0.1161	-0.3470	0.7285
previous induced abortion	0.9971	0.9659	1.0293	0.0162	-0.1762	0.8600
previous spontaneous abortion	1.0111	0.9773	1.0460	0.0173	0.6376	0.5236
education level	1.0048	0.9905	1.0194	0.0073	0.6660	0.5053
parental income	1.0217	1.0063	1.0375	0.0077	2.7702	0.0056
country	0.8910	0.8285	0.9582	0.0371	-3.1095	0.0018
region Sjelland	1.1140	1.0710	1.1586	0.0200	5.3831	<0.0001
region Syddanmark	1.1609	1.1212	1.2019	0.0177	8.4215	<0.0001
region Midtylland	1.1222	1.0840	1.1618	0.0176	6.5216	<0.0001
region Nordjylland	0.9524	0.9103	0.9965	0.0230	-2.1085	0.0349
mother with disorder	1.1832	1.1535	1.2137	0.0129	12.9661	<0.0001
father with disorder	1.1469	1.1086	1.1866	0.0173	7.9067	<0.0001

Neoplasms - benign	exp(coef)	LCL	UCL	SE	Z	Pr(Z)
surgery	1.1587	1.0666	1.2588	0.0422	3.4856	0.0004
paternal age	0.9816	0.9641	0.9995	0.0092	-2.0077	0.0446
maternal age	0.9747	0.9548	0.9950	0.0105	-2.4324	0.0149
gestation length	1.0037	0.9895	1.0182	0.0072	0.5197	0.6032
maternal bleeding	1.0461	0.9950	1.0998	0.0255	1.7672	0.0771
fetal oxygen deprivation	1.0648	0.8167	1.3884	0.1353	0.4643	0.6424
pregnancy oedema	1.0048	0.9019	1.1194	0.0550	0.0876	0.9301
apgar5 score	1.0140	0.9589	1.0723	0.0284	0.4900	0.6240
birth weight	1.0129	0.9994	1.0265	0.0068	1.8771	0.0605
preexisting hypertension	0.8439	0.5743	1.2402	0.1964	-0.8638	0.3876
preexisting diabetes	0.9857	0.7747	1.2542	0.1228	-0.1166	0.9071
previous induced abortion	1.0086	0.9749	1.0435	0.0173	0.4962	0.6197
previous spontaneous abortion	1.0309	0.9941	1.0690	0.0185	1.6430	0.1003
education level	1.0104	0.9949	1.0262	0.0079	1.3162	0.1880
parental income	1.0174	1.0007	1.0343	0.0084	2.0545	0.0399
country	0.9182	0.8503	0.9914	0.0391	-2.1786	0.0293
region Sjelland	1.1023	1.0565	1.1502	0.0216	4.4958	<0.0001
region Syddanmark	1.1711	1.1281	1.2157	0.0190	8.2857	<0.0001
region Midtylland	1.1363	1.0947	1.1794	0.0190	6.7241	<0.0001
region Nordjylland	0.9155	0.8713	0.9620	0.0252	-3.4945	0.0004
mother with disorder	1.2190	1.1832	1.2560	0.0152	12.9987	<0.0001
father with disorder	1.1771	1.1253	1.2312	0.0229	7.1015	<0.0001

Circulatory - all	exp(coef)	LCL	UCL	SE	Z	Pr(Z)
surgery	1.1095	0.9915	1.2416	0.0573	1.8113	0.0700
paternal age	0.9728	0.9497	0.9965	0.0122	-2.2423	0.0249
maternal age	0.9449	0.9193	0.9712	0.0140	-4.0395	<0.0001
gestation length	0.9718	0.9533	0.9906	0.0098	-2.9144	0.0035
maternal bleeding	1.1090	1.0394	1.1832	0.0330	3.1312	0.0017
fetal oxygen deprivation	0.7509	0.4983	1.1314	0.2091	-1.3694	0.1708
pregnancy oedema	1.0297	0.8959	1.1834	0.0709	0.4124	0.6799
apgar5 score	1.2205	1.1388	1.3080	0.0353	5.6408	<0.0001
birth weight	0.9711	0.9538	0.9887	0.0091	-3.1903	0.0014
preexisting hypertension	1.0808	0.7285	1.6035	0.2012	0.3863	0.6992
preexisting diabetes	0.7387	0.5184	1.0525	0.1806	-1.6764	0.0936
previous induced abortion	0.9789	0.9347	1.0252	0.0235	-0.9018	0.3671
previous spontaneous abortion	1.0458	0.9958	1.0982	0.0249	1.7941	0.0727
education level	0.9683	0.9484	0.9887	0.0106	-3.0222	0.0025
parental income	0.9742	0.9529	0.9959	0.0112	-2.3185	0.0204
country	0.8303	0.7509	0.9181	0.0512	-3.6260	0.0002
region Sjelland	0.9899	0.9363	1.0465	0.0283	-0.3574	0.7207
region Syddanmark	0.9141	0.8692	0.9613	0.0256	-3.4932	0.0004
region Midtylland	0.9490	0.9031	0.9972	0.0252	-2.0703	0.0384
region Nordjylland	0.7810	0.7309	0.8345	0.0338	-7.3074	<0.0001
mother with disorder	1.5195	1.4543	1.5875	0.0223	18.7128	<0.0001
father with disorder	1.3424	1.2880	1.3989	0.0210	13.9763	<0.0001

Nervous System - all	exp(coef)	LCL	UCL	SE	Z	Pr(Z)
surgery	1.2274	1.0726	1.4045	0.0687	2.9793	0.0028
paternal age	0.9847	0.9554	1.0148	0.0153	-0.9999	0.3173
maternal age	0.8797	0.8493	0.9111	0.0178	-7.1625	<0.0001
gestation length	0.9900	0.9659	1.0147	0.0125	-0.7974	0.4252
maternal bleeding	1.1289	1.0393	1.2262	0.0421	2.8752	0.0040
fetal oxygen deprivation	1.4049	0.9544	2.0680	0.1972	1.7237	0.0847
pregnancy oedema	1.2854	1.1030	1.4981	0.0781	3.2149	0.0013
apgar5 score	1.0320	0.9378	1.1357	0.0488	0.6459	0.5183
birth weight	0.9797	0.9575	1.0024	0.0116	-1.7483	0.0804
preexisting hypertension	0.8768	0.4556	1.6871	0.3339	-0.3936	0.6938
preexisting diabetes	1.4043	0.9900	1.9921	0.1783	1.9037	0.0569
previous induced abortion	1.0529	0.9935	1.1157	0.0295	1.7424	0.0814
previous spontaneous abortion	1.0954	1.0300	1.1650	0.0314	2.9014	0.0037
education level	0.8994	0.8759	0.9235	0.0134	-7.8557	<0.0001
parental income	0.9365	0.9104	0.9633	0.0143	-4.5560	<0.0001
country	0.7145	0.6249	0.8169	0.0683	-4.9182	<0.0001
region Sjelland	1.4321	1.3367	1.5342	0.0351	10.2147	<0.0001
region Syddanmark	1.1997	1.1245	1.2799	0.0330	5.5163	<0.0001
region Midtylland	1.0251	0.9588	1.0959	0.0340	0.7277	0.4667
region Nordjylland	0.7665	0.6995	0.8399	0.0466	-5.7004	<0.0001
mother with disorder	1.5669	1.4606	1.6809	0.0358	12.5365	<0.0001
father with disorder	1.4492	1.3301	1.5789	0.0437	8.4835	<0.0001

Mental - all	exp(coef)	LCL	UCL	SE	Z	Pr(Z)
surgery	1.2268	1.1612	1.2961	0.0280	7.2937	<0.0001
paternal age	1.0091	0.9976	1.0207	0.0058	1.5607	0.1185
maternal age	0.9133	0.9011	0.9257	0.0068	-13.2301	<0.0001
gestation length	0.9846	0.9752	0.9941	0.0049	-3.1486	0.0016
maternal bleeding	1.1319	1.0965	1.1685	0.0162	7.6431	<0.0001
fetal oxygen deprivation	1.1633	0.9659	1.4011	0.0948	1.5946	0.1107
pregnancy oedema	1.0867	1.0103	1.1690	0.0372	2.2352	0.0254
apgar5 score	1.0616	1.0236	1.1011	0.0186	3.2139	0.0013
birth weight	0.9671	0.9584	0.9759	0.0046	-7.2049	<0.0001
preexisting hypertension	1.1412	0.9160	1.4216	0.1121	1.1780	0.2387
preexisting diabetes	1.1409	0.9881	1.3172	0.0733	1.7974	0.0722
previous induced abortion	1.1335	1.1092	1.1583	0.0110	11.3430	<0.0001
previous spontaneous abortion	1.0746	1.0488	1.1010	0.0123	5.8058	<0.0001
education level	0.9628	0.9527	0.9731	0.0054	-6.9779	<0.0001
parental income	0.7814	0.7726	0.7904	0.0058	-42.4237	<0.0001
country	0.4989	0.4733	0.5259	0.0268	-25.9091	<0.0001
region Sjelland	0.8569	0.8332	0.8812	0.0142	-10.8170	<0.0001
region Syddanmark	0.9141	0.8924	0.9365	0.0123	-7.2892	<0.0001
region Midtylland	0.7747	0.7553	0.7945	0.0129	-19.7626	<0.0001
region Nordjylland	0.5596	0.5397	0.5803	0.0185	-31.3700	<0.0001
mother with disorder	2.0114	1.9642	2.0598	0.0121	57.6536	<0.0001
father with disorder	1.6000	1.5555	1.6457	0.0143	32.6849	<0.0001
